# dSPRINT: predicting DNA, RNA, ion, peptide and small molecule interaction sites within protein domains

**DOI:** 10.1093/nar/gkab356

**Published:** 2021-05-17

**Authors:** Anat Etzion-Fuchs, David A Todd, Mona Singh

**Affiliations:** Lewis-Sigler Institute for Integrative Genomics, Princeton University, Carl Icahn Laboratory, Princeton, NJ 08544, USA; Department of Computer Science, Princeton University, 35 Olden Street, Princeton, NJ 08544, USA; Lewis-Sigler Institute for Integrative Genomics, Princeton University, Carl Icahn Laboratory, Princeton, NJ 08544, USA; Department of Computer Science, Princeton University, 35 Olden Street, Princeton, NJ 08544, USA

## Abstract

Domains are instrumental in facilitating protein interactions with DNA, RNA, small molecules, ions and peptides. Identifying ligand-binding domains within sequences is a critical step in protein function annotation, and the ligand-binding properties of proteins are frequently analyzed based upon whether they contain one of these domains. To date, however, knowledge of whether and how protein domains interact with ligands has been limited to domains that have been observed in co-crystal structures; this leaves approximately two-thirds of human protein domain families uncharacterized with respect to whether and how they bind DNA, RNA, small molecules, ions and peptides. To fill this gap, we introduce dSPRINT, a novel ensemble machine learning method for predicting whether a domain binds DNA, RNA, small molecules, ions or peptides, along with the positions within it that participate in these types of interactions. In stringent cross-validation testing, we demonstrate that dSPRINT has an excellent performance in uncovering ligand-binding positions and domains. We also apply dSPRINT to newly characterize the molecular functions of domains of unknown function. dSPRINT’s predictions can be transferred from domains to sequences, enabling predictions about the ligand-binding properties of 95% of human genes. The dSPRINT framework and its predictions for 6503 human protein domains are freely available at http://protdomain.princeton.edu/dsprint.

## INTRODUCTION

Domains are recurring protein subunits that are grouped into families that share sequence, structure and evolutionary descent (1–5). Protein domains are ubiquitous—over 95% of human genes contain complete instances of ∼6000 Pfam domain families (1)—and can be identified from sequence alone. Domains facilitate a wide range of functions, among the most important of which are mediating interactions. Knowing the types of interactions each domain enables—whether with DNA, RNA, ions, small molecules or other proteins—would be a great aid in protein function annotation. For example, proteins with DNA- and RNA-binding activities are routinely analyzed and categorized based upon identifying domain subsequences (6,7), and the functions of signaling proteins can be inferred based upon their composition of interaction domains (8). Nevertheless, not all domains that bind these ligands are known, and new binding properties of domains continue to be elucidated experimentally (9).

While identifying the types of molecules with which a protein interacts has already proven to be an essential component of functional annotation pipelines (10), pinpointing the specific residues within proteins that mediate these interactions results in a deeper molecular understanding of protein function. For example, protein interaction sites can be used to assess the impact of mutations in the context of disease (11,12), to identify specificity-determining positions (13) and to reason about interaction network evolution (14). Recently, it has been shown that domains provide a framework within which to aggregate structural co-complex data and this per-domain aggregation allows accurate inference of positions within domains that participate in interactions (15). Domains annotated in this manner can then be used to identify interaction sites within proteins, and such domain-based annotation of interaction sites has been the basis of approaches to detect genes with perturbed functionalities in cancer (16) and to perform systematic cross-genomic analysis of regulatory network variation (17).

In this paper, we aim to develop methods to comprehensively identify, for all domains found in the human genome, which types of ligands they bind along with the positions within them that participate in these interactions. To date, computational methods to characterize domain–ligand interactions (15,18–20) have relied heavily on structural information. However, structural data are not readily available for all domain families, with only a third of the domain families with instances in human protein sequences observed in any co-complex crystal structure (see Supplementary Methods S1.1.3).

For the remaining domain families, current techniques fail to identify whether they are involved in mediating interactions, what types of ligands they bind, and the specific positions within them that participate in binding. A method that can identify a domain’s ligands and interaction positions without relying on structural information not only would be a great aid in characterizing domains of unknown function (DUFs) or other domains for which interaction information is unknown but also would enable further downstream analyses by identifying interaction sites within proteins that contain instances of these domains. Indeed, at present, the proteins for only ∼13% of human genes have co-complex structures with ligands and from which interaction sites can be derived (see Supplementary Figure S1).

Here we develop a machine learning framework, **d**omain **S**equence-based **PR**ediction of **INT**eraction-sites (dSPRINT), to predict DNA, RNA, ion, small molecule and peptide binding positions within all protein domains found in the human genome. While previous methods have predicted sites and regions within protein sequences that participate in interactions (e.g., (21–25), and see reviews (26,27)), dSPRINT instead predicts the types of ligands a domain binds along with the specific interaction positions within it, even for domains that lack any structural characterization. dSPRINT’s focus on predicting the ligand-binding properties of domains is complementary to existing approaches that annotate interaction sites within protein sequences. To train dSPRINT, we leverage InteracDome (15), a large dataset of domains for which binding information has been annotated based on the analysis of co-complex crystal structures, while considering a comprehensive set of features extracted from a diverse set of sources. Our framework aggregates information at the level of protein domain positions, and features for each domain position are derived from sites corresponding to this position across instances within protein sequences. Site-based attributes considered include, for example, physicochemical properties of amino acids, predicted secondary structure, conservation across homologs and allelic frequency across populations. By jointly considering features derived from multiple analogous positions across proteins, our approach can amplify signals that are useful for pinpointing binding positions within domains.

Our framework dSPRINT trains five different classifier models (gradient boosting, random forests, logistic regression, support vector machines and neural networks) to predict which positions within domains bind five different types of ligands (DNA, RNA, ion, small molecule and peptide) and then utilizes a novel heterogeneous ensemble architecture that combines information across different ligand types. Using rigorous cross-validation testing, we empirically demonstrate excellent performance in identifying ligand-binding positions within domains and additionally show that dSPRINT’s per-position predictions can be utilized effectively to identify which types of ligands a domain is binding. Finally, we apply dSPRINT to 4,286 human domains for which ligand-binding information is not known (15), and newly predict whether they bind DNA, RNA, ions, small molecules or peptides.

## MATERIALS AND METHODS

### Identifying protein domains and creating our training set

We first curate a training set of human protein domains with reliably annotated ligand-binding positions. We begin by identifying all Pfam (1) domains with matches in human genes. This corresponds to 6029 protein domain families that collectively cover 95% of human genes (see Supplementary Figure S1). Then, to construct our training set, we restrict to domains that (i) have >10 instances in the human genome to leverage aggregating information across human genes at the domain level, (ii) have per-position binding labels (15) as described in the next section and (iii) pass certain domain similarity thresholds to avoid trivial cases where multiple near-identical domains are utilized. Supplementary Methods S1.1.1 describes the details of this process including how we identify domain instances using hidden Markov Model (HMM) profile models from the Pfam-A (version 31) database (1) using HMMER (28), and Supplementary Methods S1.1.2 describes the domain similarity filtering done using hhalign (v3.0.3) from the HH-suite (29). Ultimately, we obtain a training set of 44,872 domain positions within 327 domains.

### Defining structure-based training labels within domains

Domain positions are labeled as binding RNA, DNA, ion, peptide and small-molecule based on InteracDome (15), a collection of binding positions across 4128 protein domain families annotated via analysis of PDB co-complex crystal structures. For a position within a domain, its InteracDome positional score corresponds to the fraction of BioLip (30) structural co-complexes in which the amino acid found in that domain position is within 3.6Å of the ligand. For each InteracDome domain–ligand type pair annotated to have instances in at least three non-redundant PDB structures, we define positive (i.e., binding) and negative (i.e., non-binding) positions using the pair’s InteracDome-defined threshold, which corresponds to precision in predicting ligand-binding positions within individual proteins in an internal cross-validation setting. We use a threshold of 0.75 for ion and a threshold of 0.5 for all other ligands. We choose these thresholds to balance the trade-off between having enough training data versus the frequency with which that position is observed to participate in interactions across different structures. For each domain–ligand type pair, all positions with InteracDome scores greater than or equal to the domain-binding threshold are labeled as positives, and all the positions with scores equal to 0 are labeled as negatives. The positions with scores below the domain threshold and above 0 are not used in our training, as they are not consistently observed to participate in binding ligands across domain instances and small values may correspond to artifacts within some co-complex structures. Importantly, for each position within a domain that is labeled as a positive for a particular ligand type, there is an instance of that domain within a solved co-crystal structure where the amino acid in that position is in contact with the ligand type. Similarly, for each position within a domain that is labeled as a negative for a particular ligand type, there is no instance of that domain within any solved structure where the amino acid in that position is found to be in contact with the ligand type. Table 1 summarizes the ligands’ InteracDome precision thresholds, and the number of positives and negatives that we used to train, test, and develop the final machine learning framework.

**Table 1.** Summary of the training set

Ligand	InteracDome threshold	Number of binding domains	Number of positives	Number of negatives
RNA	0.5	21	247	43 720
DNA	0.5	33	397	43 884
Ion	0.75	91	351	39 630
Peptide	0.5	72	436	41 105
Small molecule	0.5	132	825	32 697

For each ligand type, we use the specified InteracDome (15) threshold to define binding (i.e., positives). Positions with an InteracDome score of 0 are deemed non-binding (i.e., negative). We list here the number of binding domains, the number of positives and the number of negatives we use in the dSPRINT training.

### Feature vector construction

**Figure 1.**
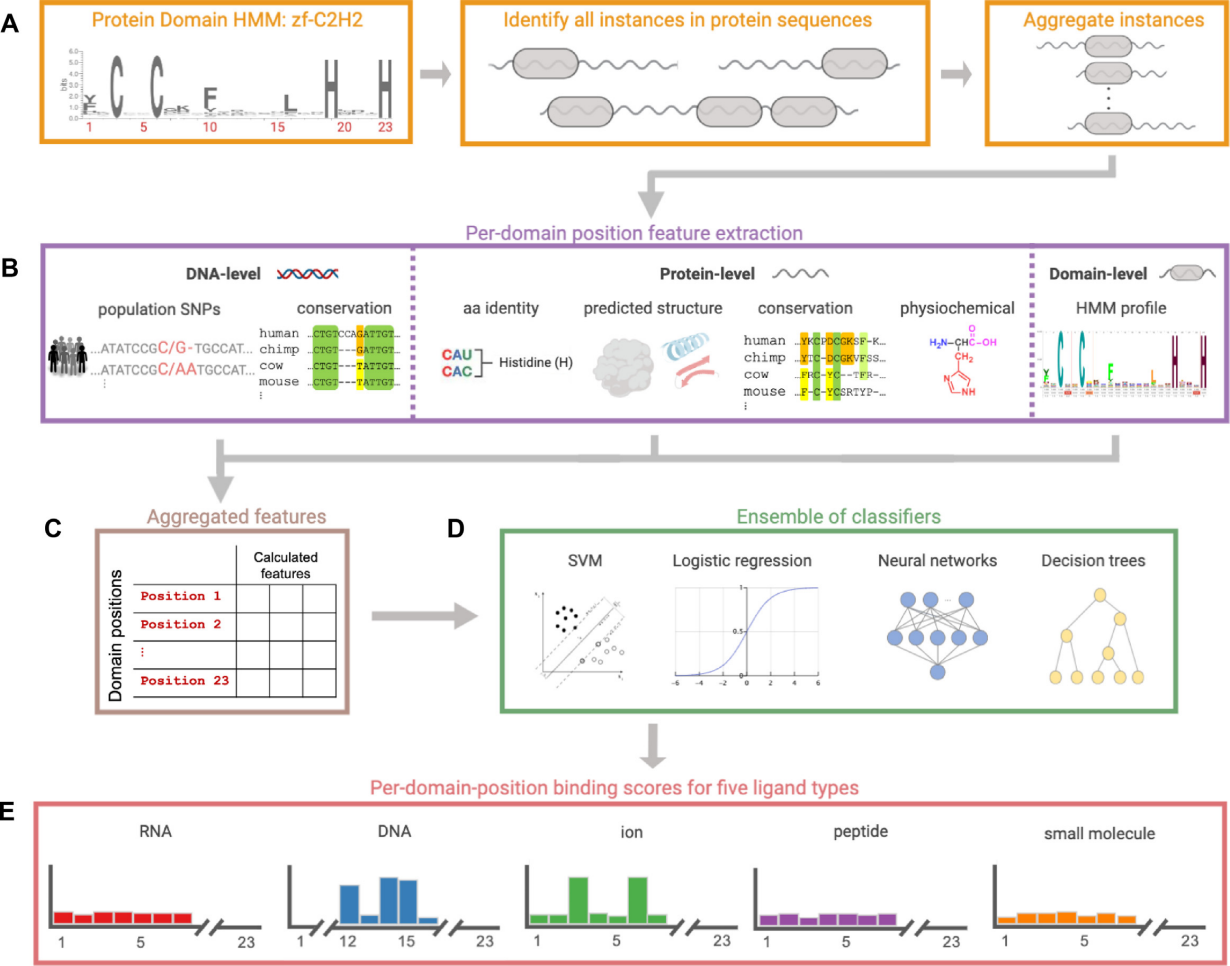
Workflow for predicting binding positions in protein domains. (**A**) For a given protein domain (represented as an HMM), instances of the domain are found across all human proteins and aggregated to construct per-position features. The zf-C2H2 domain (PF00096) is shown as an example. (**B**) For instances of the domain, features for each position are calculated at either the DNA base, protein amino acid or whole-domain level. The figure illustrates a few example features calculated at each level. DNA-level features (left) include population allele frequencies and evolutionary conservation. Protein amino acid-level features (middle) include amino acid identity, information derived from predicted structure (e.g., secondary structure and solvent accessibility), amino acid conservation across orthologs, and physicochemical properties of the amino acids. Domain-level features (right) include the HMM emission probabilities and the predominant amino acid at each position. (**C**) Features from the three levels are aggregated across instances for each protein domain position. (**D**) Using these features and a set of known DNA, RNA, ion, peptide and small molecule binding positions within domains, we train a heterogeneous ensemble of classifiers to identify positions binding each ligand type. (**E**) Results from these classifiers are combined and each final model outputs per-domain-position binding scores for one of the ligand types (Figure generated using biorender.com).

For each protein domain position, we create feature vectors as follows. Features arise from aggregating information from instances of the domains in human genes. Instances of each domain are found within human proteins via HMMER. Then, for each protein domain position, as defined by Pfam HMM match states, we aggregate diverse information (as described below) for all corresponding protein sites (Figure 1A, i.e., those that map to that domain position within instances of the domain). For each protein, we use the canonical UniProt (v2018_05) isoform if it is available, and otherwise, the longest isoform is used.

Our features can be divided into three groups based upon the level at which the features are calculated and aggregated: DNA-level features, which are aggregated by base across codons whose amino acids map to the domain position; protein amino acid-level features, which are aggregated according to domain positions; and protein domain-level features which do not require further aggregation (Figure 1B). The features calculated can also be subcategorized into six functional groups: (i) conservation-based features using scores from PhastCons (31) and PhyloP (32), as well as amino acid conservation (33) and Pfam emission probabilities, (ii) features that are based upon physicochemical properties and the identity of amino acids (e.g., hydrophobicity, charge, functional group, volume, hydrogen bonds and secondary structure propensity), (iii) population features based on variants and their observed frequencies in the ExAC database (34), as well as predicted functional effect scores for each non-synonymous variant (SIFT (35), Polyphen (36), and Clinvar (37)) as provided by ExAC, (iv) features based upon sequence-based structure prediction using SPIDER2 (38), (v) features based upon the location of a position within the domain and the length of its containing domain and proteins and (vi) measures of selective pressures on protein residues in a given position (e.g., dN/dS (39)). Some features are also calculated by summing the values of individual position features across windows that are centered at the protein domain position of interest. Altogether, our feature vector has size 753, as many of our feature types are represented via multiple dimensions (e.g., the identity of amino acids is represented via a one-hot encoding). Of the 753 features, 320 are windowed features and 433 are the original per-position features. A detailed list of features and how they are extracted and encoded is provided in Supplementary Methods S1.3 and Supplementary Table S1.

### Machine learning methods tested

For each of our five ligands, we use the labeled domain positions and their feature vectors to train five separate binary classifiers to predict positions within domains that contact them: (i) logistic regression (LR), (ii) support vector machines (SVM) with the RBF kernel, (iii) random forest (RF), (iv) gradient boosting as implemented by the XGBoost algorithm (40) (XGB) and (v) neural networks (NN) (Figure 1C). The LR, SVM and RF classifiers are built using scikit-learn (41) and NNs are built using Pytorch. We chose these classification methods as they are among the most popular and have complementary strengths. Data for LR, SVM and NN are *Z*-score normalized based on just the training partition prior to training and testing. Altogether, we obtain 25 different trained ‘base’ models (five for each ligand). Details about the versions of the algorithms used, along with parameter settings and architectures are given in Supplementary Table S2.

### Heterogeneous ensemble architectures combining different ligands

We also develop stacking-based approaches to combine our base models. Stacking is an ensemble learning technique that combines multiple models via a meta-classifier or a meta-regressor where the base-level models are trained on the training set, and then a meta-model is trained using the outputs of the base level models as features (42). We consider four architectures of two-layer stacking (Supplementary Figure S2). For each of them, the first layer includes a subset of our 25 trained base machine-learning models, and the second layer consists of outputs of these base models with or without the original features. We refer to the ensemble architectures according to the number of base models (M) and the number of ligands (L) present in the architecture, and whether the original features (F) are added to the features table (as indicated by true (t) or false (f)). Our architectures are: (i) ‘M5L1F*t*’: all five base models trained on the target ligand including the original features, (ii) ‘M1L5F*t*’: one base model, XGB, trained on all the ligands (and not just the target ligand) including the original features, (iii) ‘M5L5F*t*’: all the base models trained on all the ligands including the original features (i.e., all the available features and probabilities from the first layer are used), and (iv) ‘M5L5F*f*’: all the base models trained on all the ligands but using just their outputs and not the original features. For the second layer model (i.e., the meta-model), we primarily use XGB since we have found that it is the best-performing model in the first layer across all the ligands. However, since XGB can be unstable when used with a small number of features, we use LR as the meta-model for the M5L5F*f* architecture that does not use the original features. Altogether, for each of the four stacking architectures, we obtain five trained ensembles, where each predicts per-domain-position binding scores for one of the five different ligand types (Figure 1D).

### Per-domain multi-label stratification across all ligands

We perform 5-fold cross-validation, where labels for all ligand types are balanced simultaneously across the folds. Additionally, all examples arising from the same domain are kept together in one fold to avoid information leakage when constructing windowed features that use information from neighboring amino acids. To accomplish this, we extend the IterativeStratification algorithm (43) with the added constraint of keeping examples of the same group (in our case positions of a domain) within the same fold. See Supplementary Methods S1.6.1 for a detailed description. We note that we performed domain filtering to get a non-redundant set of domains (see Materials and Methods subsection ‘Identifying protein domains and creating our training set’), and thus domains in the same or different folds are not similar to each other, and cross-validation performance is not over-estimated as a result of domain similarity. The sizes of the folds, along with the number of examples of each type in each fold, are given in Supplementary Figure S3.

### Evaluation criteria

We evaluate the performance of our classifiers with respect to: (i) identifying positions across all domains that bind the ligand of interest (global performance) and (ii) identifying binding positions within a domain known to bind a particular ligand (per-domain performance). For both tasks, we measure performance by computing the precision-recall (PR) curve, the receiver operator curve (ROC), the area under the precision-recall curve (AUPRC), and the area under the receiver operator curve (AUC). We use AUPRC as our primary performance metric as ROC curves can overestimate the performance of a classifier on imbalanced data. Since AUPRC is sensitive to test sets with different positive rates, we also consider AUPRC as compared to the expected performance by a classifier that predicts that each example is a positive or negative uniformly at random; that is, we divide the measured AUPRC by the fraction of binding positions for that ligand. We refer to this as the AUPRC fold-improvement (AUPRC-FI).

For each ligand type, when we measure global performance, we consider binding and nonbinding positions for that ligand across all domains. When we measure per-domain performance, we consider binding and non-binding positions for each specific domain and ligand pair. These per-domain measures can be calculated only for domain–ligand pairs that have at least one binding (positive) position.

For each model, we train it using each subset of four folds, and compute its AUPRC, AUC and AUPRC-FI on the fifth unseen fold; for our performance evaluations, we report the average of these values over the five test folds.

### Hyperparameter tuning

Our models are tuned and selected based upon performance on the global prediction task as measured by AUPRC. Hyperparameters are optimized using the ‘random search’ approach (44) where hyperparameter combinations are randomly sampled from a predefined hyperparameter search space (see Supplementary Methods S1.4.2). We note that because our dataset is imbalanced (with many more negative examples than positive examples), we include as a hyperparameter a weight that penalizes errors on positives more than errors on negatives. The complete list of hyperparameters and the way they are sampled are summarized in Supplementary Table S2.

We use nested 5-fold cross-validation for tuning hyperparameters for all of our base models. That is, hyperparameter tuning is performed using validation sets consisting of folds within the training set only. See Supplementary Methods S1.4.3 for a detailed description of the tuning procedure for the base models. We train our stacked models with an additional internal cross-validation within our nested cross-validation procedure (45), as out-of-fold predictions from the base models are needed for the stacking models. See Supplementary Methods S1.4.4 for a detailed description of the tuning and training of the stacking models.

### Predicting whole-domain binding functionality

We train five additional XGB models that predict whether a domain as a whole binds each of the five considered ligand types. Here, a domain is considered to bind a ligand if it has at least one position that participates in an interaction with that ligand (see Table 1) and is considered not to bind a ligand if all of its positions are non-binding (i.e., have InteracDome scores of 0 for that ligand). Note that using this definition, some domains are unclassified with respect to a ligand. The features input to these XGB models are derived from the outputs of the trained per-domain-position classifiers. Because domains have different lengths, we summarize both the per-domain-position predictions and domain features into a fixed number of features (e.g., maximum predicted per-domain-position binding score and mean conservation across the domain). Additionally, we add a feature that captures spatial attributes of the domain positions by counting the number of windows of length five within the domain that have at least two positions with a predicted score above a certain threshold. See Supplementary Methods S1.5 for a detailed description of the whole-domain features.

We train each model using 5-fold cross-validation. To prevent data leakage and obtain out-of-fold predictions, features corresponding to per-domain-position binding predictions for the training folds are obtained from classifiers trained to make per-position predictions using the other three training folds. Similarly, for each test fold, the features corresponding to per-domain-position binding predictions are computed using the other four folds. We use the same hyperparameter tuning random search approach described above and evaluate model performance using AUPRC.

### Comparison of dSPRINT to adapting methods for predicting interaction sites within protein sequences

To the best of our knowledge, there are no other methods for predicting ligand-binding positions within domains or predicting whether a domain as a whole binds a particular ligand type. However, there are methods that do a different but related task where they predict which positions within proteins bind these ligands. These methods can be adapted to make predictions about domain positions, as described below, and the performances of these adapted methods can be compared to dSPRINT. Similar to (46), we use the following criteria to pick methods for inclusion in our comparative analysis: (i) the method must be available either as a working webserver or as source code; (ii) the method must be explicit about which data were used in its training set, as otherwise, we cannot know if the domains we are testing it on are similar to sequences that were present in its training set; (iii) the method must generate per-position binding predictions; and (iv) there must be a large enough test set once we consider domains that are not in our training dataset and that are not in the training set of the other method (as described below). Supplementary Table S3 gives the methods we considered for inclusion (obtained from two recent review papers (26,46)). Based on our selection criteria, we are able to compare dSPRINT to two methods for predicting nucleic acid binding sites, Dr PIP (21) and DRNApred (22), and two methods for predicting peptide binding sites, SCRIBER (47), and SPPIDER (48). To assess these methods in the context of per-domain-position predictions, we derive per-domain-position scores from their amino acid site-level predictions. In particular, for a given domain, we obtain all protein sequences within human that contain that domain, and for each position within the domain, we average the scores from each method across all the protein positions aligned to that domain position. We stress that these tools were not designed for the task of predicting binding positions within domains and that there might be different ways to use them to obtain per-domain-position predictions; we are simply trying to obtain reasonable baseline predictions using existing approaches.

To create a valid benchmark test set that does not overlap the training sets of dSPRINT, and the other methods, we first construct a set of all protein domains with InteracDome labels that have at least two but at most 10 instances in human, as these are not used by dSPRINT for training. We note that due to the relatively small number of instances of these domains, dSPRINT may be disadvantaged as it relies on aggregating features from across domain instances. Next, we remove domains that are either similar to one another or similar to domains in dSPRINT’s training set (using the same HHalign similarity thresholds used for constructing our training set, see Supplementary Methods S1.1.2). For each of the remaining domains, of the human sequences that contain it, to create our nucleic acid test set and peptide test set, we remove those sequences that are similar to any protein in the Dr PIP training set and SCRIBER training set, respectively (i.e., have a BLAST *E*-value <0.5 to any protein in the training set and have at least 30% sequence identity across the length of the BLAST alignment, as recommended by the Dr PIP sequence similarity guidelines). This removes all the sequences for many domains and ultimately yields five domain families that bind DNA within seven proteins with 727 total labeled domain positions, seven domain families that bind peptide within eight proteins with 1522 total labeled domain positions, and two domain families that bind RNA within two proteins. Due to the small number of RNA-binding positions in this set, we only consider performance with respect to DNA- and peptide-binding positions. Additionally, we do not make any further adjustments to remove proteins overlapping with the DRNApred or SPPIDER training sets, as this removed too many sequences and domains.

## RESULTS

### Excellent performance in identifying ligand-binding positions within domains

**Figure 2.**
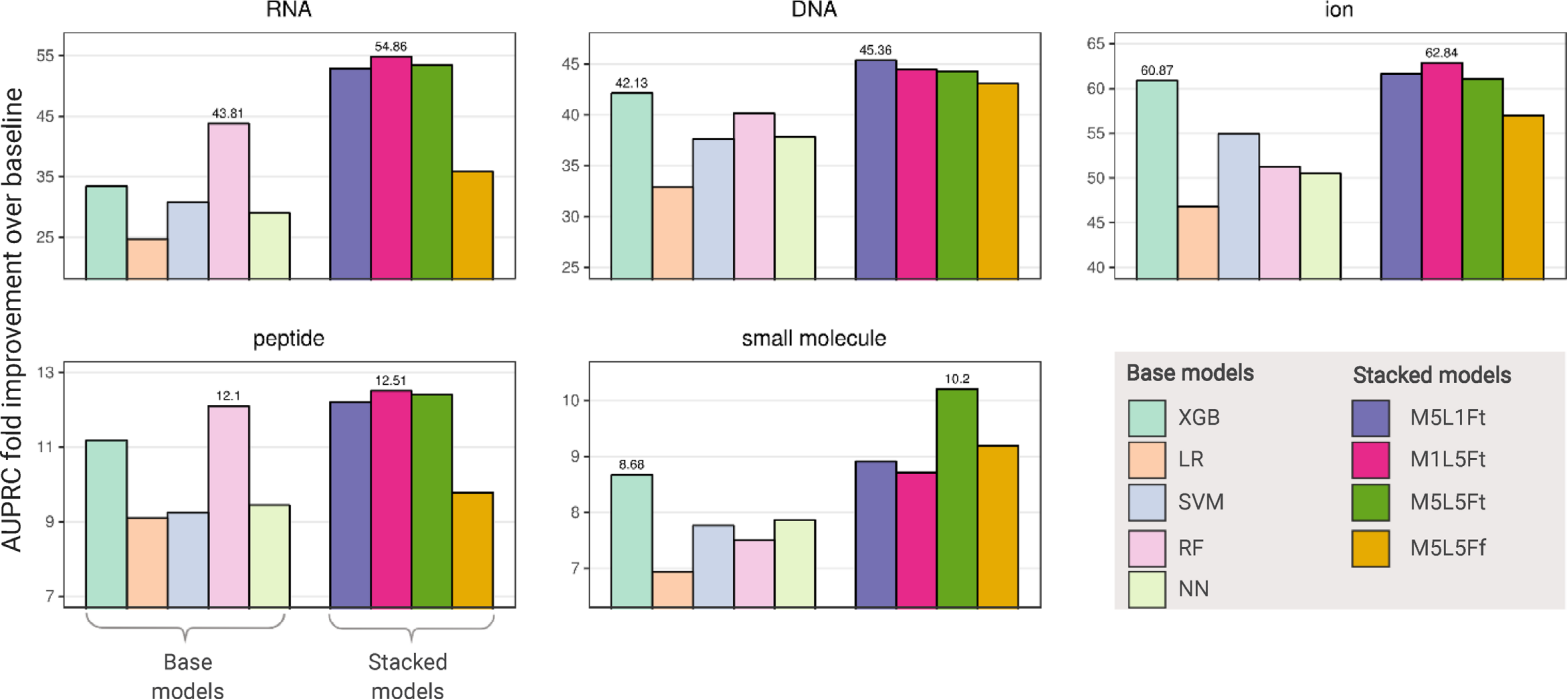
Performance in predicting ligand-binding positions across all domains. For each of the five ligands, we use 5-fold nested cross-validation to assess performance of nine trained classifiers. For each ligand, for each of the five base models and the four stacked models, we calculate the mean AUPRC for each test fold. Then, we obtain the AUPRC-FI for each fold by dividing the AUPRC by a baseline corresponding to the fraction of binding positions of that ligand in the fold. Average fold improvements across the test folds are given. For each ligand, AUPRC-FIs are listed for the best performing base and stacked models.

We first train and evaluate the predictive performance of each of our models using 5-fold cross-validation. We compute the mean over all the folds of the AUPRC-FI (Figure 2), as well as the full PR (Supplementary Figure S4) and ROC (Supplementary Figure S5) curves. For each ligand type, the best performing models have very high AUPRC-FIs, with values of 62.84, 54.86, 45.36, 12.51 and 10.2 for ion, RNA, DNA, peptide and small molecule, respectively. The corresponding AUCs for these models are also high (Supplementary Figure S5), and are >0.9 for RNA, DNA and ion, and >0.8 for small molecule and peptide. Overall, we find that machine learning methods are highly effective in identifying ligand-binding positions within domains.

### Novel ML stacking architecture critical for high predictive performance

For each ligand type, while there is at least one base model with >8 fold improvement of the AUPRC as compared to baseline, a stacked model yields the best AUPRC-FI (Figure 2). Among the base models, the decision tree models XGB and RF are outperforming the others across all ligands, with XGB obtaining the best performance for DNA, ion and small molecule and RF obtaining the best performance for RNA and peptide; the second layers in our stacking models are thus based on XGB.

For four of five ligand types (RNA, ion, peptide and small molecule), the best performing ensemble includes base models trained on other ligands (i.e., M1L5Ft or M5L5Ft). For these ligands, knowledge about whether a position is predicted to participate in binding to other ligand types improves the prediction beyond what is obtained using the standard stacking approach of combining different models trained on the same prediction task (represented by M5L1F*t*). We note that for all five ligand types, the three stacked architectures that use the original features in addition to different combinations of the outputs of the base models (i.e., M5L1Ft, M1L5Ft and M5L5Ft) outperform all the base models. Moreover, for all ligands, these three ensembles outperform M5L5Ff, an ensemble that uses only the first-layer outputs, thereby demonstrating a clear advantage for using the original features. Nevertheless, the first layer predictions are by themselves very informative, as M5L5Ff performs similarly to the other ensembles for DNA and, to a lesser extent, ion.

Across ligand types, the best stacking approach can yield significant improvements in performance. The biggest performance improvement for a stacking approach over the base models is seen for RNA, where the best stacking model improves the AUPRC by 25%, with the biggest performance improvement at the beginning of the PR curve (Supplementary Figure S4). Stacking also considerably improves the performance in predicting small molecule binding positions, where the best stacking model improves the AUPRC by 17%. Stacking results in notable but more modest improvements for the other three ligands (7%, 3%, and 3% for DNA, ion and peptide, respectively). Altogether, we find our stacking architecture that integrates different models and ligands to be important for obtaining high performance in predicting ligand-binding positions within domains.

### Final dSPRINT model chosen for each ligand

For each ligand, we choose the architecture that has the best performance as judged by the log-fold change in AUPRC as compared to baseline performance. This is M1L5F*t* for RNA, ion, and peptide, M5L1F*t* for DNA, and M5L5F*t* for small molecule. For the remainder of the paper, we will use these models when we refer to dSPRINT.

### dSPRINT’s top predictions for most ligands are highly accurate

**Figure 3.**
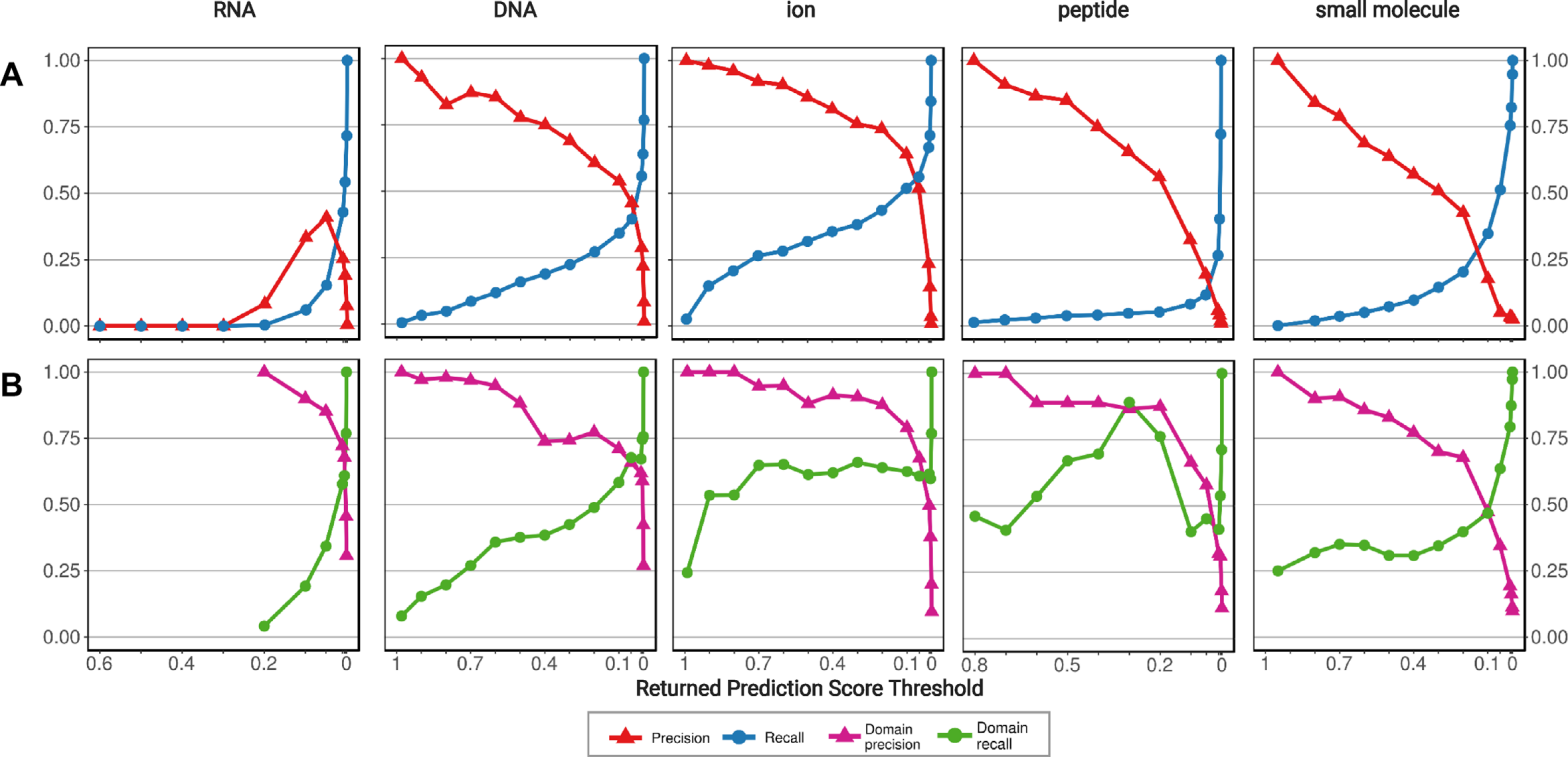
Precision and recall as a function of prediction scores. For the best performing model for each ligand, we plot the precision and recall when considering all predictions greater than a specific score threshold (*x*-axis). Predictions are made in cross-validation, and predictions across all folds are considered together. (**A**) Precision (red) and recall (blue) averaged across the five folds. (**B**) Precision (pink) and recall (green) values computed for each of the domains that bind the ligand and then averaged across all such domains. All precision and recall are plotted up to the highest prediction score threshold returned for each ligand.

We next evaluate cross-validation performance as a function of the score returned by the best performing dSPRINT model for each ligand type since users of dSPRINT might be interested in considering only the highest and most reliable predictions. For DNA, ion, peptide and small molecule, positions with the highest predicted scores are true positives a large fraction of the time, as reflected by the high precision at high predicted score thresholds (Figure 3A). For example, for ion, 92% of the positions with a predicted score greater than 0.7 are predicted correctly as binding, and this accounts for 26.4% of all the ion binding positions. For DNA, peptide and small molecule, we see similar trends where we have high precision at high score thresholds that decreases as we lower the threshold and the recall increases. Moreover, for all four of these ligand types, there is a threshold for which dSPRINT has 100% precision on our dataset: 0.98 for DNA, 0.99 for ion, 0.8 for peptide and 0.95 for small-molecule. For RNA, the trend is different, as no predicted score threshold guarantees high precision; this is due to 11 of the highest scoring 12 positions arising from two domains for which we have no observed RNA-binding functionality. While these predictions could be false positives, they could also represent functionalities that are not observed in our structurally derived InteracDome labels.

### dSPRINT’s top predictions within domains are reliable

We next evaluate how well our chosen models predict binding positions within a domain known to bind a specific ligand type (i.e., the domain has positive examples that allow us to compute per-domain metrics) (Figure 3B). As expected, across all five ligand types, for the same score thresholds, within-domain mean precisions (pink lines) are generally greater than or equal to the precisions obtained when trying to identify binding positions across all domains (compare to Figure 3A). Similarly, the within-domain mean recalls (light blue lines) are greater than or equal to the recalls for the global task. Even for RNA, we find that binding positions within known RNA-binding domains are predicted with high accuracy, as reflected by a domain precision of 1 at a prediction score threshold of 0.2.

When we consider only the highest scoring position for each domain, this top prediction is correct for between 40% and 67% of domains in each of the ligand types (Supplementary Table S4). When considering the five highest predictions in each domain, at least one of these five is a binding position for between 67% and 88% of the domains binding each of the ligand types (Supplementary Table S4); in fact, across all the ligand types together, the five top predictions are all correct for more than 10% of the domains. Overall, we find excellent performance in identifying specific binding positions within a domain if we know the type of ligand it is binding.

### dSPRINT achieves high performance for most domains

**Figure 4.**
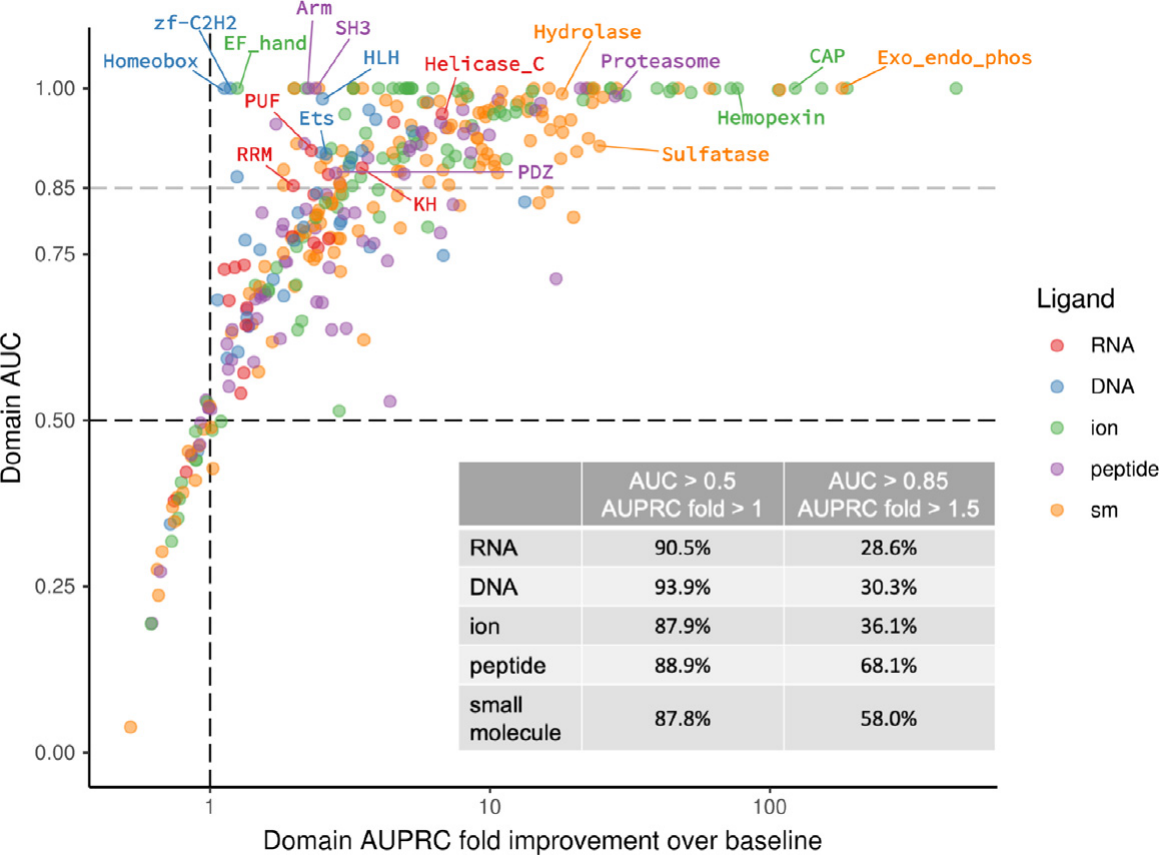
Cross-validation performance evaluation on individual domains. For each domain–ligand pair, the domain AUC (*y*-axis) is plotted against the domain AUPRC-FI (*x*-axis). The fold change is computed between the actual AUPRC and a baseline AUPRC corresponding to the fraction of the binding positions within the domain. The vast majority of the domain–ligand pairs (∼90%) have performance exceeding that of the random baselines (black dashed lines) of AUC=0.5 and AUPRC-FI=1, and about a third achieve performance above AUC=0.85 and AUPRC-FI =1.5, as noted in the table for each ligand type. There are 184 domain–ligands pairs with AUCs >0.85 (grey dashed horizontal line), with some examples of these labeled.

We next consider performance for each domain–ligand pair individually (Figure 4) as measured by domain-level AUC and AUPRC-FI. Even though our models are tuned to optimize the global AUPRC metric and not the per-domain metrics, the vast majority of the binding domains for all the ligand types have AUCs >0.5 and AUPRC-FIs >1, and more than a quarter of the domains for each ligand type achieve AUC > 0.85 and AUPRC-FI > 1.5. Most of the cases where our models fail to accurately rank within-domain positions involve domains with low fractions of positives—domains with AUC < 0.5 have 5% positives on average—and where all positions tend to be predicted with low binding scores.

There are many domains for which our predictions lead to near perfect performance with AUCs close to 1, including the RNA-binding domains Pumilio (PF00271), RRM (PF00076), KH (PF00013) and Helicase (PF00271); the DNA-binding domains Homeobox (PF00046), HLH (PF00010), zf-C2H2 (PF13984) and Ets (PF00178); the ion-binding domains CAP (PF00188), EF_hand (PF13833) and Hemopexin (PF00045); the peptide-binding domains SH3 (PF00018), PDZ (PF00595), Arm (PF00514) and Proteasome (PF00227); and the small molecule-binding domains Hydrolase (PF00702), Exo_endo_phos (PF03372) and Sulfatase (PF008844). Indeed, for 45 domain–ligand type pairs, we achieve perfect ranking with AUCs equal to 1.0. For several ion- and small molecule-binding domains that are relatively long and yet have a small number of binding positives, our predictions obtain greater than a 100-fold improvement over what would be expected by a baseline method that ranks positions arbitrarily.

### Per-domain-position predictions can be used to predict the binding activity of domains

**Figure 5.**
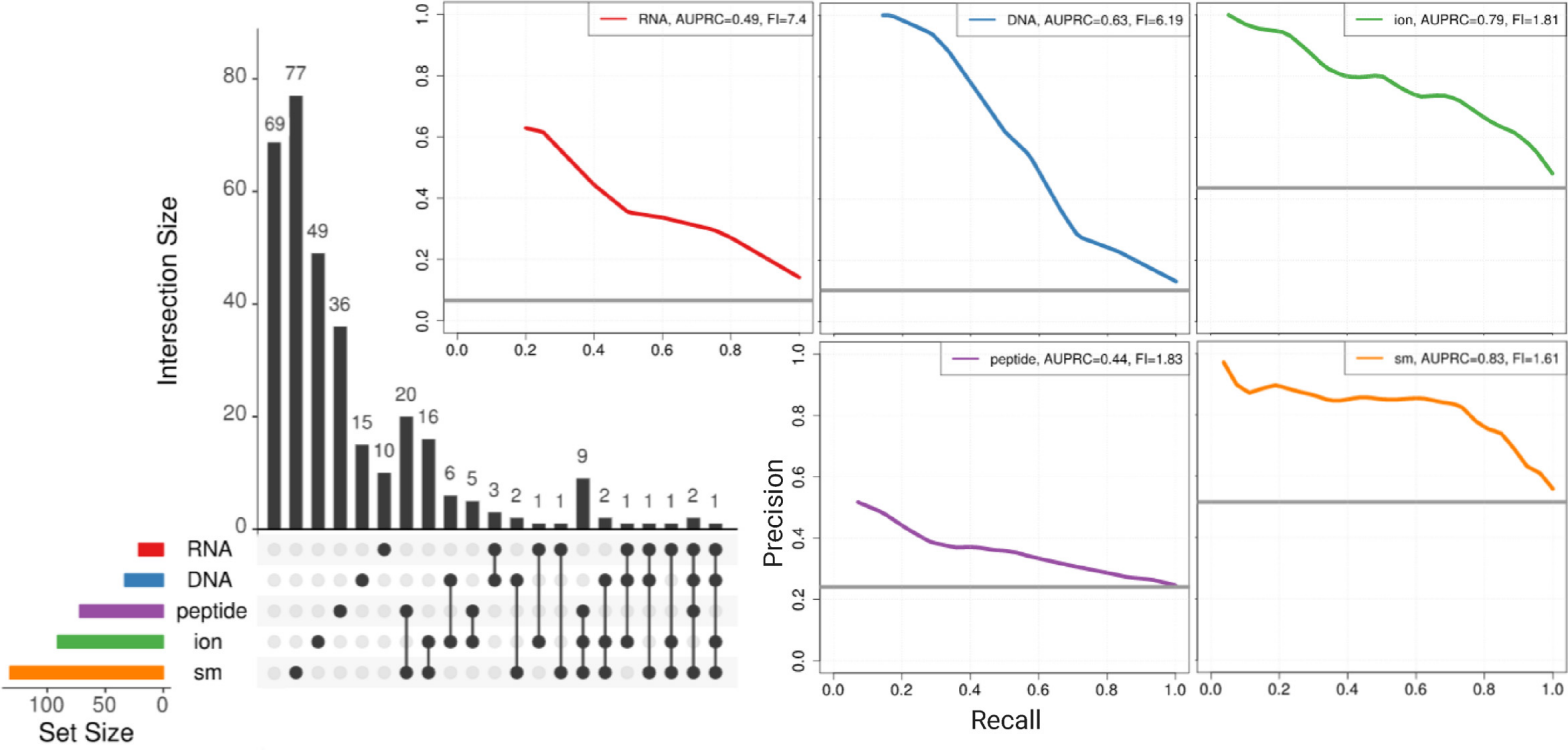
Performance in predicting the ligand type a domain binds. PR curves for predicting whether a domain binds a ligand using 5-fold cross-validation. For each ligand type, the grey horizontal line represents the random baseline corresponding to the fraction of positives in the training dataset. For each ligand, AUPRC and AUPRC-FI are given in an inset on the top-right of the panel. An UpSet plot (67) on the left shows the numbers of domains binding each of the ligands (i.e., the sizes of the training sets) as horizontal bars. The sizes of the intersections between the sets of domains known to bind these ligands are shown with a vertical bar plot.

For many domains, the ligands they bind (if any) are unknown. For this reason, we next train an XGB machine learning model that uses dSPRINT’s per-domain-position predictions of ligand binding to predict whether a domain as a whole binds DNA, RNA, peptides, small molecules or ions. We use 5-fold cross-validation on a training set of domains known to bind a particular ligand and show PR curves for each of the five ligands (Figure 5). For DNA, ion and small molecule, we achieve perfect or near-perfect precision for our highest ranked binding domains and have AUPRCs of 0.62, 0.79 and 0.83, respectively. The biggest AUPRC-FIs are achieved for predicting whether domains bind RNA or DNA (7.39 and 6.18 AUPRC-FIs, respectively). Peptide, ion, and small molecule have lower AUPRC-FIs of 1.84, 1.72 and 1.60, respectively. Overall, we find that our per-domain-position predictions of binding activity can be used effectively to predict binding interactions at the level of the entire domain.

### Performance of dSPRINT as compared to adapting methods for predicting interaction sites within proteins

We next compare dSPRINT, trained using all the data (see Supplementary Methods S1.4.5 for a detailed description of how the final models are trained), to existing approaches for similar tasks. While we know of no other methods that predict the ligands or ligand-binding positions of domains, there are several methods that predict whether sites within proteins are involved in interactions. We can aggregate their predictions by domain to obtain predictions for whether domain positions bind ligands (see ‘Materials and Methods’ section). We compare dSPRINT to aggregating results from two approaches for predicting DNA-binding sites within protein sequences, Dr PIP (21) and DRNApred (22), and two approaches for predicting peptide-binding sites within proteins, SCRIBER (47) and SPPIDER (48). On carefully constructed independent benchmark datasets of annotated DNA- and peptide-binding positions within domains (see ‘Materials and Methods’ section), we find for DNA that, as compared to aggregating predictions from Dr PIP or DRNApred, dSPRINT has more than twice the global AUPRC (i.e. when considering predictions for all positions across the dataset together) as well as substantially higher average per-domain AUPRC (Table 2 and Supplementary Figure S6). dSPRINT also has higher AUC than the other methods across the entire dataset, and higher average per-domain AUC than DRNApred but similar though lower average per-domain AUC than Dr PIP. For peptide, as compared to aggregating predictions from SCRIBER or SPPIDER, dSPRINT’s AUPRC measurements are substantially higher: dSPRINT’s global AUPRC is 3-fold higher, and its average per-domain AUPRC is 2-fold higher than the other methods (Table 2 and Supplementary Figure S7). dSPRINT also has a higher global AUC and average per-domain AUC than the other methods. Overall, dSPRINT is much better at predicting DNA- and peptide-binding positions within domains than baseline approaches based on aggregating predictions from existing state-of-the-art methods for predicting interaction sites within proteins.

**Table 2.** Comparative performance for predicting DNA- and peptide-binding positions within domains

Ligand	Method	AUPRC	AUC	Mean domain AUPRC	Mean domain AUC
DNA	dSPRINT	**0.5450**	**0.8853**	**0.3866**	0.7618
	Agg. Dr PIP	0.2697	0.8219	0.2696	**0.7843**
	Agg. DRNApred	0.1262	0.7176	0.2011	0.7175
peptide	dSPRINT	**0.1522**	**0.6836**	**0.2345**	**0.6331**
	Agg. SCRIBER	0.0409	0.5256	0.0867	0.4265
	Agg. SSPIDER	0.0545	0.4668	0.1255	0.4606

dSPRINT’s performance on independent benchmark datasets of 727 DNA-binding domain positions and 1,522 peptide-binding domain positions as compared to baseline methods that aggregate, by domain position, per-amino acid DNA or peptide-binding predictions made by state-of-the-art methods for predicting interaction sites within protein sequences. For all methods, AUPRC and AUC is computed based on positions across all domains together, and the mean per-domain AUPRC and AUC is computed based on considering positions in each domain separately and then averaging. For three out of four measures, dSPRINT outperforms aggregating DNA-binding scores by either DRNApred or Dr PIP, and for all four measures dSPRINT outperforms aggregating peptide-binding scores by either SCRIBER or SPPIDER.

### Predicting ligand-binding properties for domains of unknown function

We next use dSPRINT, trained using all the data, to predict binding activity for functionally uncharacterized Pfam DUF domains. To highlight our top predictions (Supplementary Table S5), we set score thresholds corresponding to a global recall of 0.4 for RNA and DNA and a global recall of 0.5 for ion, peptide, and small molecule (corresponding to precisions of 0.33, 0.75, 0.77, 0.39 and 0.57 for RNA, DNA, ion, peptide and small molecule, respectively). Of the 467 DUFs in human that do not overlap any of our training datasets, 6, 10, 15, 15 and 33 of them have prediction scores above these thresholds for RNA, DNA, ion, peptide and small molecule, respectively.

Several of the proteins containing these domains have annotations consistent with our whole-domain predictions. For example, DUF1087 and DUF4074 are predicted by dSPRINT to bind DNA, and are found in CHD and Hox proteins respectively, and both of these protein families are known to bind DNA (49,50). Similarly, dSPRINT predicts that DUF1897 binds RNA, and it is found in FUBP1, a multifunctional DNA- and RNA-binding protein (51).

We also use our trained models to predict specific binding positions within the DUFs (all DUF per-domain-position predictions are available on the dSPRINT website). Four of dSPRINT’s top 10 predictions of RNA-binding positions are found in DUF1754, which is the only domain found in FAM32A, a known RNA-binding protein (52,53). For peptide-binding, the top three positional predictions are within DUF1986, and while this domain is not included in our training dataset (due to its small number of instances in the human proteome), it is involved in peptide binding and InteracDome’s highest per-position peptide-binding scores correspond to those that are most highly ranked by dSPRINT.

Our predictions also yield some interesting hypotheses about the functions of human proteins. For example, DUF1220 (also known as the Olduvai domain) is found in NBPF proteins and is notable for its lineage-specific expansions in human, its correlation with brain size, and its putative association with neurological disorders such as autism (54–56). While the functions of this domain and the NBPF proteins are unknown, dSPRINT predicts that DUF1220 binds DNA, which is consistent with the intriguing proposal that NBPF proteins could be transcription factors (57).

## DISCUSSION

Here we have trained machine learning models to predict which positions within domains are involved in interactions with DNA, RNA, ion, peptides or small molecules. Additionally, these per-domain-position predictions are themselves used to predict whether a domain as a whole binds one of these ligands. Our new domain-centric approach enables the characterization of numerous human domains for which no proteins that contain them have solved co-complex structures. Since >95% of human proteins contain domains (Supplementary Figure S1), by transferring predictions from domains to the sequences within which they are found, we can uncover ligand-binding regions and positions across the human proteome.

We have designed dSPRINT using primarily non-linear classifiers, which excel at discovering higher-order interactions between features. Moreover, since different machine learning models can capture different aspects of a dataset, we have implemented a heterogeneous stacking architecture that not only combines predictions across models but also across different ligand types. We have empirically demonstrated that stacked models improve predictive power for all ligands (Figure 2). Notably, in four of the five ligands, the best performing stacking architecture uses predictions from classifiers trained on the other ligand types, suggesting that information on other binding potential helps predict the specific type of ligand bound.

Binding positions for some ligands appear to be more difficult to predict than for others. This can be due to either biological or technical challenges (or both). For example, ion-binding positions often consist of conserved amino acids of certain types (58), whereas small molecule-binding positions as a group represent a diverse array of interactions of different amino acids with different molecule types. These biological differences, which lead to a more diverse set of features for small molecule-binding positions, make small molecule-binding positions more difficult to predict than ion-binding positions. In the case of RNA, we have fewer examples of binding positions (Table 1), which translates to a greater data imbalance and a harder prediction task. This is further complicated by the fact that RNA-binding proteins may recognize shape as opposed to making sequence-specific interactions. dSPRINT’s stacking architecture seems to mitigate some of these biological and technical difficulties; for example, the stacked models result in approximately 17% and 25% improvement as compared to the base models for predicting small molecule- and RNA-binding positions respectively.

By examining the final machine learning models using feature importance techniques, it is possible to glean which features are most important for predicting different types of ligand-binding positions within domains. In preliminary analysis using the XGBoost trained models’ built-in feature importance (based on the ‘Gini Importance,’ as implemented by scikit-learn (41)), we observe that conservation and structural features are important for predicting all types of ligand-binding positions. Other features are more important for predicting certain types of ligand-binding positions than others, thereby suggesting critical aspects of various types of interactions. For example, the features most important for the model for predicting DNA-binding positions include information on hydrogen bonds, charge and the probabilities for positively charged amino acids; this is consistent with the fact that negatively charged DNA is bound by positively charged amino acids. In contrast, as compared to the models for predicting other types of ligand-binding positions, the model for predicting peptide-binding positions within domains relies heavily on population-level selection features and relatively less on physiochemical features; this may be due to the large physicochemical variability in these types interactions.

As with all supervised learning approaches, our approach relies upon a training set of positive and negative examples. We utilize labels that are extrapolated from curated co-complex structures. While interactions that are observed in these co-complex structures are highly reliable, structural data has inherent biases due to the experimental procedures (30). For instance, certain types of proteins are harder to crystallize than others, and even when a co-complex protein structure can be experimentally determined, information about the various interactions that a protein makes may be incomplete. For example, some protein-RNA interactions happen only in response to certain physiological and environmental cues (59), which can make it difficult to characterize these interactions *in vitro*. Further, a large fraction of RNA-binding proteins do not have a known RNA-binding domain (60). Examining our cross-validation performance, dSPRINT identifies several RNA-binding positions within domains that are not labeled as binding RNA, which causes low precision in our high-scoring RNA-binding positions (Figure 3A). While some predictions are likely false positives, others may correspond to RNA-binding domains that are not observed in our structurally derived training sets. For example, several positions within the TFIIS domain are scored highly for binding RNA; this domain is not labeled as RNA-binding and is instead found in co-complex structures with DNA. However, the protein TCEA1 that contains the domain TFIIS is reported as RNA-binding by six studies (59). Moreover, the TFIIS domain is found in the TFIIS gene that is critical for transcript elongation and has been shown to be involved more broadly in nucleic acid binding as part of the RNA polymerase II machinery (61). Overall, there is some evidence that some of our false positive RNA-binding predictions may actually be involved in mediating interactions with RNA.

While we have demonstrated the usefulness of our predictions for annotating DUF domains, our framework can be used for annotating many types of other protein domains, including assigning novel functions for already annotated domains. Moreover, knowledge of the specific sites within domains involved in interactions can help prioritize disease-causing mutations (16,62) and may result in identifying novel drug targets, as most drugs operate by competing for ligand-binding sites (63).

Future applications might extend our approach beyond the human proteome. For instance, the ability to annotate ligand-binding properties of domains across the evolutionary spectrum may advance our understanding of the functional impact of the evolution of protein domain architectures (64,65). Our approach may also be applicable in metagenomics, where many observed gene families do not have significant sequence similarity to experimentally characterized domains or sequences (66). Techniques such as dSPRINT that uncover binding properties of domains may prove useful for functionally annotating HMM profiles built from these gene families.

To conclude, we expect that dSPRINT will be a great aid for expanding our knowledge of domain functions and molecular interactions, for annotating the molecular functions of proteins, and for performing numerous domain-based analyses.

## DATA AVAILABILITY

dSPRINT source code and trained models are available at: http://github.com/Singh-Lab/dSPRINT.

## Supplementary Material

gkab356_Supplemental_File
