# Supplementary material for: dSPRINT: predicting DNA, RNA, ion, peptide and small molecule interaction sites within protein domains
Source: Nucleic Acids Res. 2021 May 17;49(13):e78. doi: 10.1093/nar/gkab356 (PMC8287948; doi:10.1093/nar/gkab356)
Supplement: gkab356_Supplemental_File [file gkab356_supplemental_file.pdf]

# Supplementary Information for:

# dSPRINT: predicting DNA, RNA, ion, peptide and small molecule interaction sites within protein domains

Anat Etzion-Fuchs, David A. Todd, and Mona Singh

**Supplementary methods** **1**

**1 Supplementary methods** **1**

1.1 Data collection and preprocessing of protein domains . . . 1
1.1.1 Identifying protein domains across human genes . . . 1
1.1.2 Filtering similar domains . . . 1
1.1.3 Calculating the structural coverage of human domains and proteins . . . 1
1.2 Data collection and preprocessing for features . . . 2
1.2.1 Processing genomic conservation scores . . . 2
1.2.2 Processing sequence-based structural predictions . . . 2
1.2.3 Processing human variation data . . . 2
1.3 Data representation and feature construction . . . 3
1.3.1 Conservation-based features . . . 3
1.3.2 Physicochemical features . . . 4
1.3.3 Structurally-predicted features . . . 5
1.3.4 Population variant-based features . . . 6
1.3.5 Selection-based features . . . 7
1.3.6 Location and length-based features . . . 9
1.3.7 Windowed features . . . 9
1.4 Machine learning methods . . . 9
1.4.1 Base-level predictors . . . 9
1.4.2 Hyperparameter search space . . . 10
1.4.3 Hyperparameter tuning for the base models . . . 10
1.4.4 Training and hyperparameter tuning for the stacked models . . . 11
1.4.5 Model selection and final dSPRINT model training . . . 11
1.5 Whole-domain feature construction . . . 11
1.6 Algorithm Extensions . . . 13
1.6.1 Iterative Group Stratification . . . 13
1.6.2 Frequency-based pN/pS . . . 13

**2 Supplementary figures** **20**

**3 Supplementary tables** **27**

# 1 Supplementary methods

## 1.1 Data collection and preprocessing of protein domains

### 1.1.1 Identifying protein domains across human genes

We first identified protein domains that are found in the human proteome as follows. We downloaded all $104,763$ known and predicted human protein isoforms for $23,393$ genes from the Ensembl database (build GRCh37, release 86) [1]. We considered the subset of $92,065$ isoforms from $20,805$ genes for which the genomic DNA sequences matched the cDNA sequence and the cDNA sequence translated to the protein sequence with up to $5\%$ sequence mismatch. We searched for all instances of the $16,712$ Hidden Markov Models from the Pfam-A database (version 31) [2] using HMMER (v2.3.2 and v3.1b) [3]. We considered only domain instances that pass Pfam's default gathering thresholds, have residues at the first and last domain's positions, and have the most likely residue at highly-conserved positions (i.e., those positions with Pfam emission probability $\geq 0.99$). This process yielded $6,029$ protein domain families with matches within $19,823$ genes in human. To build our training set, we focus on the 701 protein domains that have more than 10 instances across human genes.

### 1.1.2 Filtering similar domains

We next pruned our set of 701 domains to remove highly sequence similar domains as follows. We assume that domains that are not found in a Pfam clan are not sequence similar to other domains. For the 442 domains within clans, we computed similarities to all other domains in the same Pfam clan using hhalign (v3.0.3) from HH-suite [4]. Of $1,279$ pairs of domains for which similarity was computed, 367 pairs were considered similar using the criteria recommended by the hhalign software and userguide [4] as they have in both directions: (1) p-value $\leq 0.001$ (2) percent of matching aligned column pairs $\geq 20\%$, and (3) raw similarity score normalized by the number of aligned columns $\geq 0.6$. These domain pairs involved 152 domains and we built a graph where each of these domains is a vertex and there are edges between domains found to be similar. Edges between vertices are weighted as follows. First, for each pair of vertices, we considered the raw similarity scores normalized by the number of aligned columns computed for both directions of hhalign runs, as suggested in the hhalign userguide for a similarity measurement. Next, we converted these to distances by subtracting from the maximum normalized similarity score of 1, and for each pair of vertices, we took the smaller of the values as the edge distance. There were 36 connected components in the graph, each of which can be considered a similarity group. The mediode of each connected component, computed as the vertex with the smallest average shortest path to all other vertices, was taken to represent the group of domains; this removed 116 domains and left a total of 585 domains, a subset of which have InteracDome labels as to which positions are involved in binding ligands.

### 1.1.3 Calculating the structural coverage of human domains and proteins

To calculate the percent of human domain families that have at least one instance in a solved structure in complex with DNA, RNA, small molecule, ion, or peptide, we used the InteracDome collection of *Representable Interactions* (https://protdomain.princeton.edu/interacdome_download). This collection is derived from the PDB [5] and BioLip [6] and corresponds to domain-ligand interactions for which there is at

least one co-complex structure in the PDB. $2,229$ of $6,029$ human protein domain families have at least one instance in the *Representable Interactions* set. This corresponds to $36.9\%$ of the human domain families that have an instance in some co-complex structure in the PDB; note that these structures could involve sequences from any organism (not just human).

To calculate the percent of human genes that have solved structures in complex with DNA, RNA, small molecules, ions, or peptides, we used the Biomart Python package to map BioLip (April 2020) entries to human genes. Of the $102,558$ PDB IDs within Biolip, sequences within $15,629$ of them mapped to $2,698$ human gene ENSEMBL IDs. This corresponds to $13\%$ of the human genes that are present in some solved structure interacting with a ligand (see Supplementary Figure S1 for the total number of human genes).

## 1.2 Data collection and preprocessing for features

We compute features for our machine learning models based on human variation data, sequence-based structure predictions, and conservation values. In this section, we describe how we obtain and process some of these data from existing resources.

### 1.2.1 Processing genomic conservation scores

We downloaded the conservation scores PhastCons [7] and PhyloP [8] from the UCSC genome browser (version hg19, 100-way vertebrate multiple alignment). PhyloP measures conservation for individual columns, whereas PhastCons estimates the probability that each nucleotide belongs to a conserved element and smooths the scores across DNA regions. We use both measures as each provides useful information, and the two complement each another.

### 1.2.2 Processing sequence-based structural predictions

We ran the tool SPIDER2 [9] on each human protein sequence. SPIDER2 is an iterative deep-learning neural network that predicts several structural properties from sequence including secondary structure, surface accessibility, and backbone angles. We ran SPIDER2 on the full protein sequences even though we use just the information calculated for positions within protein domain instances because SPIDER2 considers the full protein sequence to make its predictions.

### 1.2.3 Processing human variation data

We downloaded natural variants occurring in the exomes of 60,706 healthy individuals from The Exome Aggregation Consortium (ExAC, release 0.3)[10]. ExAC consists of observed genomic variants and their observed frequencies across all the individuals sequenced in VCF format, accompanied by sequencing coverage information. It also includes SIFT [11], Polyphen [12], and Clinvar [13] scores for each non-synonymous alteration; we extracted this information as well. A few filters suggested by the ExAC authors to retrieve high-quality variants were applied: a PASS filter status by Genome Analysis Toolkit (GATK)[14] Variant Quality Score Recalibration (VQSR)[15]; individuals with depth (DP) $\geq 10$; individuals with genotype quality (GQ) $\geq 20$; and excluding variants located in one of the 10 1-kb multi-allelic (quad-allelic or higher) regions of the genome as defined in [10]. Additionally, only exome regions with mean coverage $\geq 20$ were considered. This process yielded 42,464,073 high-quality variants.

We uncovered all ExAC single nucleotide variants occurring within protein domain instances and determined the variant effect (i.e., synonymous or non-synonymous). For each protein position, we defined the major allele as the most frequent amino acid at that position according to ExAC; we note that this may differ from the amino acid in the Ensembl reference.

## 1.3 Data representation and feature construction

For each protein domain position, we extract and encode a common set of features. Here we discuss the details of computing and representing the features in our framework. For sections 1.3.1-1.3.5, for each domain, we consider all proteins and corresponding gene sequences with instances of that domain, compute amino acid- or DNA-level attributes, and then aggregate these attributes by domain position (i.e., by match state). Note that some features are also used as windowed features (further discussed in section 1.3.7) and are labeled with ($w$). Table S1 summarizes our features.

### 1.3.1 Conservation-based features

We calculated conservation-based features at three different levels: (1) DNA, (2) amino acid, and (3) domain.

**DNA-level features.** For each domain position, we considered the codons that correspond to the amino acids that match that position across all instances of the domain under consideration. For each of the three DNA positions, PhastCons and PhyloP$^{(w)}$ conservation scores are averaged (i.e., the mean conservation at the first position of the codons, the mean conservation at the second position of the codons, and the mean conservation at the third position of the codons). We also encoded these conservation values as histograms that reflect the meaning of scores for each of the scoring methods. For PhastCons, the histogram included lower resolution for the non-conserved score region with three bins between 0 and 0.75 as well as higher resolution for the conserved score region with five bins between 0.75 and 1. For PhyloP, the histogram included two bins for negative scores that reflect accelerated evolution, relatively lower resolution for lowly conserved score regions with three bins between 0 and 3, and higher resolution for the highly-conserved score regions with six bins between 3.5 and 6. We encoded these histograms for each of the three codon positions individually. We also considered the conservation scores in the context of their original codon by computing the mean conservation across each codon and then encoding the means using the histogram binning just described. Additionally, we encoded features that correspond to the mean and standard deviation of the conservation scores for the whole domain for both scoring systems, resulting in an additional four features; note that these four features will be shared for all positions across a domain. Altogether, we have 86 DNA-level conservation features.

**Amino acid-level features.** For each protein sequence, amino acid conservation was computed using the UCSC genome browser 100-way vertebrate multiple alignment. For each column in the alignment, we multiplied the fraction of non-gapped residues by the Jensen-Shannon divergence (JSD) between those non-gapped residues and a BLOSUM 62 background amino acids distribution. This conservation measurement has previously been shown to highlight evolutionarily conserved functionally important positions within proteins [16]. For each domain position, we encoded the median JSD score of all the protein positions aligned to that position$^{(w)}$ and a histogram of these JSD scores with lower resolution for the non-conserved scores region with one bin between 0 and 0.5 along with higher resolution for the more conserved scores with four bins between 0.5 and 1. Additionally, we computed the JSD for a column consisting of all the major alleles

obtained from ExAC, which reflects conservation at that domain position across human instances. Altogether, we have seven amino-acid level conservation features.

**Domain-level features.** For each domain position, we encoded as features the Pfam emission probabilities for all the amino acids at that position, as well as the maximum emission probability across amino acids$^{(w)}$. Another boolean feature determined whether this position included any emission probability above 0.5 (the threshold used within Pfam to define conserved residues). This resulted in 22 features.

### 1.3.2 Physicochemical features

For each domain position, we considered all the positions within proteins that were a match for it and calculated features based upon the identities and properties of the amino acids corresponding to the major allele (as determined from ExAC alleles frequencies).

**Identity.** We used a 21-length vector representing the 20 amino acids and a stop codon, with counts for the number of times each was the major allele at an aligned protein position. Another 21-length vector is a probability vector that sums to 1 with these same counts normalized to their respective fractions. This resulted in 42 features.

**Charge.** We used information about an amino acid's charge [17] for each of the protein positions aligned to the domain position. The features encoded included counts of positively charged (K and R), negatively charged (D and E) and neutral (all the remaining) amino acids, as well as a feature representing the charge of the majority of aligned amino acids$^{(w)}$ (1 for positive, -1 for negative, or 0 for neutral). This resulted in four features.

**Functional group.** We used a classification of the amino acid side-chains to five functional groups [18] for each of the protein positions aligned to the domain position. As features, we encoded counts of amino acids according to whether they were polar (C, N, P, Q, S, T), aliphatic (A, G, I, L, M, V), aromatic (F, W, Y), negative (D, E), or positive (H, K, R). This resulted in five features.

**Hydrophobicity index.** We used Kyte and Doolittle hydrophobicity index values [19] associated with the amino acid for each of the protein positions aligned to the domain position. As features, we encoded the mean of this hydrophobicity index$^{(w)}$, as well as counts of polar (D, E, G, H, K, N, P, Q, R, S, T, W, Y) and counts of hydrophobic (A, C, F, I, L, M, V) residues. This resulted in three features.

**Volume.** We used information about an amino acid's volume for each of the protein positions aligned to the domain position. As features, we encoded the mean of the residue volume values$^{(w)}$ [20], and counts according to size categories [21] of tiny (A, C, G, S), small (D, N, P, T, V), and big (E, F, H, I, K, L, M, Q, R, W, Y). This resulted in four features.

**Hydrogen bonds.** We used information about an amino acid's potential number of hydrogen bond donors or acceptors [22]. As features, we encoded the mean number of potential hydrogen bond donors$^{(w)}$ and acceptors$^{(w)}$ across all protein positions aligned to the domain position. This resulted in two features.

**Secondary structure propensity.** We used Chou and Fasman secondary structure propensity values [23] associated with the amino acids for each of the protein positions aligned to the domain position. As features, we encoded the mean propensity values for alpha-helix$^{(w)}$, beta-sheet$^{(w)}$, and turn$^{(w)}$. We also encoded a binary vector of length three that records the majority preference of all major allele amino acids at that position (i.e., a binary 0/1 feature for the secondary structure type with the highest mean). This resulted in six features.

### 1.3.3 Structurally-predicted features

Knowledge of protein structure provides many useful properties that may be predictive of ligand-binding sites. For example, positions on a protein's surface are more likely to be involved in interactions with other molecules than those buried within the core of the protein. Since we do not have structures for all proteins, we use sequence-based predictions from SPIDER2 [9] of structural properties that can be applied to any protein sequence. SPIDER2 predicts position-specific scores for several structural properties, and for each protein position aligned to a domain position, we retrieved SPIDER2's structural predictions and encoded 31 features as described below.

**Surface accessibility.** SPIDER2 predicts the solvent accessible surface area (ASA) that measures the exposure of amino acid residues to solvent. We aggregate the ASA prediction values for each protein position aligned to the domain position. The features encoded are the mean$^{(w)}$ and standard deviation$^{(w)}$ of the ASA values.

**Contact number.** Another measure of residue exposure to solvent is contact number (CN), which counts the number of residues within a specific distance cut-off. SPIDER2 predicts two types of contact numbers $C\alpha - C\alpha$ and $C\alpha - C\beta$, where the former measures the number of $C\alpha$ atoms in other residues that are within a threshold around its $C\alpha$ and the latter measures the number of $C\beta$ atoms in other residues that are within a threshold around its $C\beta$. We aggregate these values for each protein position aligned to the domain position. The features encoded are the mean$^{(w)}$ and the standard deviation of these two contact number types across the aligned protein positions.

**Half-sphere exposure.** Half-sphere exposure (HSE) is an orientation-dependent contact number that separates the contacts to two half spheres (up and down) defined according to the $C\alpha - C\beta$ (HSE$\beta$) vector or neighboring $C\alpha - C\alpha$ vectors (HSE$\alpha$) [24]. SPIDER2 includes predictions for half spheres for both (HSE$\alpha$) and (HSE$\beta$) [25], which we aggregate for each protein position aligned to the domain position. The features encoded are mean$^{(w)}$ and the standard deviation of these four contact numbers across the aligned protein positions.

**Secondary structure predictions.** SPIDER2 returns predicted 3-state secondary structure probabilities for alpha-helix, beta-sheet, and coil. We aggregate these predictions for each protein position aligned to the domain position. The features encoded are the mean$^{(w)}$ and standard deviation of the predicted probabilities for each structural type. For every aligned protein position, the secondary structure with the highest predicted probability is the most probable one at that position. Taken in aggregation, we also encoded the most frequent probable secondary structure(s) across all aligned protein positions, as a length three binary vector with '1' representing which of the three structures has the highest predicted probability the most number of times; we note that there can be more than one '1' value.

**Backbone angles.** An alternative to a secondary structure representation of a protein structure is an angle-based representation of the backbone structure. An angle-based description offers a continuous representation of local conformations [26] rather than the discontinuous and somewhat arbitrary definition of three secondary structure states. SPIDER2 predicts the two backbone torsion angles $\phi$ and $\psi$, and $C_\alpha$-based angles $\theta$, and $\tau$ [27]. The features encoded are the mean$^{(w)}$ and the standard deviation for each of these four angles across the aligned protein positions.

### 1.3.4 Population variant-based features

We calculated population variant-based features using the allele frequencies reported in the ExAC database [10] for a cohort of healthy individuals. For every codon aligned to a domain position, we first identified the major allele (i.e., the amino acid that appears with the highest frequency, which does not necessarily match the reference genome) and all the minor alleles and the sum of their corresponding allele frequencies (i.e., the minor allele frequency, MAF). Note that we consider MAFs at the level of amino acids, and not individual nucleotides. Compared to neutral positions, we reasoned that positions involved in binding will display less variation, have lower frequencies of non-synonymous variants, and have a smaller number of different variants. We encoded several groups of features as described below.

**Variant frequencies features.** The features encoding the variant frequencies for each domain position include: the mean of MAFs across all the amino acids aligned to the domain position, the mean of MAFs across only the protein positions that had a variant$^{(w)}$, a histogram of the MAFs with higher resolution in the low-frequency region and lower resolution in the high-frequency region using the 10 bins $\{0, 0.0001, 0.0005, 0.01, 0.02, 0.04, 0.06, 0.08, 0.1, 0.2, 0.5\}$, the mean of MAFs, the mean of synonymous variant frequencies, and the mean of non-synonymous variant frequencies for each population in ExAC (i.e., African, America, East Asian, Finnish, Non-Finnish European, South Asian, and Other). This resulted in 33 features.

**Variant counts features.** The features encoding count summaries of the variants for each domain position include: the number of aligned codons with a non-synonymous variant, the same number divided by the total number of aligned codons, the number of nucleotide positions with a variant, the same number divided by the total number of nucleotides aligned to the domain position, the mean number of different amino acids across the population in one codon across codons that had a variant, and the fraction of aligned codons with at least one minor allele. This resulted in six features.

**Purifying selection indicators.** MAFs are indicative of selection acting on a site, as deleterious variants are likely to be rare. We calculated the fraction of codons with rare MAF out of all the aligned codons with minor alleles. As features, we encoded the fraction of codons with MAF smaller than $0.5\%$, $0.05\%$, and $0.005\%$. This resulted in three features.

**Non-synonymous variant distribution.** We were interested in understanding how the population MAFs are distributed across the protein positions that map to a domain position. For each of the amino acid positions that mapped to a domain position, we considered its MAF divided by the sum of the MAFs over all such amino acid positions. We then used these values to calculate for each domain position the Shannon entropy of the MAFs, divided by maximum possible entropy (i.e., the $\log$ of the number of mapped amino acid positions), as computed in Miller et al.'s pan-cancer study [28]. If all MAFs for the protein positions mapping to the domain position are 0, then the normalized entropy is set to the maximum entropy. High entropy indicates that variants are more uniformly spread across the aligned protein positions. This resulted in one feature.

**Identity of variant amino acids.** For each protein position that mapped to a domain position, we encoded the types of amino acids variants that were observed across the population. We computed four sets of such features where each set of features represents the 20 amino acids and a stop codon as described next. The first set of 21 features recorded counts of how many times each amino acid was a minor allele, and the second set of features takes these counts and divides by the sum of these counts (i.e., it encodes probabilities of the counts that sum to 1). The third set of features keeps track of the amino acids counts multiplied with the

corresponding variant frequencies, and the fourth set of features corresponds to the probabilities of these frequency-based counts. This resulted in 84 features.

**Substitution matrices.** For binding positions, if population variants are observed, we expect that the variants are more likely to be chemically similar. The commonly used substitution matrices BLOSUM62 and PAM40 capture this aspect of substitutability, with more probable substitutions having higher scores. For each domain position, we considered all protein positions that mapped to it and encoded the following features: the mean of BLOSUM62$^{(w)}$ and PAM40$^{(w)}$ scores of the variants (i.e., major allele to variant substitution score in these matrices) at the aligned position, the same mean but also weighted by the variant frequency, counts of the number of positive substitutions and negative substitutions, and the ratio of positive to negative substitution scores. This resulted in 10 features.

**Variant effect predictors.** SIFT [11], PolyPhen2 [12] and ClinVar [13] predictions are reported by ExAC for observed variants. These are tools that predict whether variants are damaging. SIFT and PolyPhen scores are numeric values. For ClinVar, ExAC supplied a clinical categorical label, that was converted to a score as follows: benign=-2, likely benign=-1, uncertain=0, likely pathogenic=1, pathogenic other=1.5, and pathogenic=2. These scores were encoded into several features for each domain position using all the amino acid positions that mapped to that domain position: for each of these three scoring systems, the mean score across all variants for all amino acid positions$^{(w)}$ and the mean weighted by the variant frequency were recorded. We also encoded several score specific features for SIFT and PolyPhen. For SIFT, additional features included counts across all protein positions of “deleterious” and “tolerated”, the ratio of these counts, and a feature representing the majority (i.e., are the majority of variants tolerated or deleterious). For PolyPhen, additional features included counts of “benign”, “possibly damaging”, and “probably damaging” as well as a feature representing the majority. This resulted in 14 features.

**Variant physicochemical transitions**. These features capture the chemical changes for variants occurring at the protein position aligned to a domain position. We expect variants in binding positions to result in smaller changes to the physicochemical properties than variants at other positions. These features are similar to our major allele physicochemical features discussed in Section 1.3.2, only here they are encoded with respect to the variants occurring at amino acid positions aligned to a domain position. We encoded the changes as the mean of differences from major allele to the variant with respect to several properties values, both weighted and unweighted by variant frequency: hydrophobicity$^{(w)}$, volume$^{(w)}$, H-bonds donors, H-bonds acceptors, and secondary structure propensities (alpha-helix, beta-sheet, gamma turn). We also created another set of features to represent functional groups movements that occur as a result of the variants (from five functional groups, listed in 1.3.2, to any one of these five groups or a stop codon). These were encoded as follows: count and frequency of major allele to variant substitutions that stayed within the same functional group$^{(w)}$ and those that moved to a different group$^{(w)}$, as well as counts of the specific transitions between the different groups resulting from the substitutions. This resulted in 48 features.

### 1.3.5 Selection-based features

Quantifying the selection pressure at each domain position can highlight binding positions with certain properties that need to be maintained across different domain instances. On the other hand, it can highlight positions that are generally conserved across different species but not across different domain instances, and these might be involved in determining the specificity of ligand interactions (i.e. they may vary between

domain instances). We now discuss these two angles on selection pressure.

**dN/dS inspired features.** The ratio of non-synonymous to synonymous variants between species (dN/dS) is a measure of selection. Similarly, this ratio calculated within a species (pN/pS) characterizes the selective constraint on a site at the level of a population [29]. To calculate this measurement per domain position, we constructed a pseudo sequence of all the residues that aligned to this position across all domain instances. Using variant frequencies from the ExAC database for these protein positions, we can calculate the pN/pS ratio for this "sequence". Such calculation usually requires as input all the sequences or a multiple sequence alignment of the proteins under consideration. See for example in the widely used phylogenetic analysis tool PAML [30]. This is not feasible when using variant information from over $60,000$ individuals. Therefore, we implemented an extension of the original pN/pS calculation, denoted frequency-based pN/pS (See Algorithm Extensions1.6.2), that uses variation frequencies as input instead of requiring the full protein sequences. For each domain position, we considered all protein positions that mapped to it and computed the number across the population of synonymous substitution per synonymous site (pS), the number of non-synonymous substitutions per non-synonymous site (pN), and their ratio $(\text{pN/pS})^{(w)}$. This resulted in three features.

**Ortholog-paralog conservation ratio.** This set of measurements is based on the notion that when considering an amino acid position important for ligand-binding specificity, it is likely to be conserved across orthologous proteins, but tends to vary across paralogous proteins [31]. Thus the ratio of the conservation between orthologs and paralogs can help identify ligand-binding positions.

In our framework, conservation across orthologs was computed in two ways: (1) median protein-level conservation of amino acids mapped to the domain position (computed using JSD on the 100-way vertebrate multiple alignment, as described in Section 1.3.1), and (2) mean of the ExAC MAFs of the amino acids mapped to the domain position as described in Section 1.3.4), which is a conservation measurement across human individuals in a population instead of across different species. We will use these conservation measures, which we refer to as *ortho JSD* and *ExAC MAF*, to compute features as described below.

For the conservation across paralogs, we treated every protein domain instance within humans as a different copy of the same ancestral domain. We computed the conservation across paralogs by measuring the conservation across the protein positions from different domain instances, aligned to the domain position. The amino acid at each protein position is the major allele as determined using ExAC variation frequencies. We computed the conservation of these paralogous positions in three ways: (1) JSD between the column of aligned protein positions and BLOSUM62 (as described in Section 1.3.1); (2) Shannon entropy (SE) of the instances column, another commonly used measure of conservation at a site [32], where we consider the distribution of the 20 amino acids and any other possibility (e.g., an 'X' codon); and (3) the fraction of positions that have an amino acid different from that which most commonly occurs in the column (i.e., this is a "domain instances minor allele frequency"). The latter two were added as two features, and we will also use these measures (which we refer to as *instances JSD*, *instances SE*, and *instances MAF* respectively) to compute additional features as described below.

We constructed features representing the ortholog-paralog conservation ratio as follows. They were constructed so that they have higher values when ortholog conservation is high and paralog conservation is low. Therefore, we expect that higher values will help to identify binding positions important for specificity. The eight features we encoded for each domain position are: (1) $\frac{\text{instances MAF}}{\text{ExAC MAF}}$, (2) $\frac{\text{ortho JSD}}{(1-\text{instances MAF})}$, (3) ortho JSD$\times$instances SE, (4) $\frac{\text{ortho JSD}}{(\log(21)-\text{instances SE})}$, (5) ortho JSD$+\frac{\text{instances SE}}{\log(21)}$, (6) $\frac{\text{instances SE}}{(1\text{ - ortho JSD})}$, (7) $\frac{\text{ortho JSD}}{\text{instances JSD}}^{(w)}$, and (8) ortho JSD - instances JSD.

Altogether, we have 10 features arising from considering ortholog-paralog conservation ratios (two for paralog conservation and eight for the ratios).

#### 1.3.6 Location and length-based features

All the above-mentioned features consider each position without its sequence context. This allows us to capture positional information. However, the global sequence context of the domain position of interest might help in establishing patterns for binding positions. To capture this context information, we looked at the location of the position within the domain and also at the location of the position within proteins that contain the domain. The domain-context features we considered are: the position within the domain, the domain length, and binary features that represent the position's location in the domain: beginning, middle, or end. The protein-context features we considered are: for each protein that contains the domain, we compute the mean of the proteins' lengths and counts that represent the domain's position in the protein (beginning, middle, or end). This resulted in nine features.

#### 1.3.7 Windowed features

Since an amino acid's function can be affected by its neighboring amino acids, we also use windowed features that are centered at each domain position. We consider four window sizes $w \in \{3, 7, 11, 21\}$, and 40 of the positional features described above, (denoted by $(w)$, see also Table S1). For each window and positional feature combination, we add two windowed positions, corresponding to the mean and standard deviation of the values of the features for all the domain positions within the window. This results in 320 features arising from these windows.

### 1.4 Machine learning methods

This section describes the specific machine learning implementations we used for the dSPRINT framework and also gives details about hyperparameter tuning and how we trained the final dSPRINT model.

#### 1.4.1 Base-level predictors

We detail here the classifiers used in the first level of our stacked architecture. Unless noted otherwise, the classifiers were used with their default parameters. The tuned parameters are listed in Table S2.

- **Logistic regression (LR).** Built using the scikit-learn Python library [33] LogisticRegression function, with L2 penalty.
- **Support Vector Machine (SVM).** Built using the scikit-learn Python library SVC function, with radial basis function (rbf) kernel.
- **Random forest (RF).** Built using the scikit-learn Python library RandomForestClassifier function.
- **Gradient boosting implemented by XGBoost (XGB).** Built using the XGBoost Python library [34] XGBClassifier function. Training of XGB included "early stopping" implemented by the Python library. The early stopping uses the evaluation metric "mean average precision" (map).

- **Neural networks (NN).** Built using the Pytorch Python library [35], and implemented as a feed-forward network with an input layer, three hidden layers, and an output layer, all fully connected. Sizes of the hidden layers were determined during hyperparameter tuning. The loss function used was cross-entropy, and batch normalization layers were used between each of the internal layers. The minibatch size is a tuned hyperparameter. Backpropagation was used for training. Training of the network includes batch normalization and dropout implemented using a normalization layer between each of the internal layers and a dropout after the last hidden layer.

### 1.4.2 Hyperparameter search space

We tuned model hyperparameters using the random search approach [36]. As described in Supplementary Table S2, most parameters are sampled from a uniform space and some from a logarithmic space since for certain parameters (e.g., learning rate and learning decay in NN) a change in the smaller range of values can have a larger effect on model dynamics.

We also used early stopping for XGB and NN to determine the number of estimators for XGB and the number of epochs for NN. For XGB, we used the built-in early stopping mechanism supplied in the XGBoost Python library [34] with the number of "early stopping rounds" equal to 500 and allowed a one time increment to 750 if the first 500 estimators did not improve the initial random performance. For NN, we implemented an early stopping strategy with dynamically updated patience as described in [37]. Briefly, initial patience is set but as training proceeds, the patience is dynamically updated: every time a new epoch is deemed an "improvement epoch," its number is added to the initial patience. We used initial patience of 100 and defined an epoch as improving if its validation AUPRC is higher than a previous AUPRC (i.e., tolerance of 0).

We randomly sampled 100 hyperparameter combinations, and each for combination, the mean AUPRC across the four validation folds was computed. We then chose the combination with the highest mean AUPRC, excluding combinations with high AUPRC but random (close to 0.5) AUC in at least one fold. The chosen combination was used in the outer 5-fold cross-validation to compute the performance for the respective test fold.

### 1.4.3 Hyperparameter tuning for the base models

We use nested five-fold cross-validation for tuning hyperparameters for all of our base models. An inner four-fold cross-validation is used to tune the parameters and select the best model (i.e., one of the four folds is used in turn as a validation set, while a model is trained on the other three folds), and the outer fold is used to evaluate the model selected by the inner cross-validation. For each of the five test folds, we sample 100 hyperparameter combinations and choose the combination with the highest average validation performance across the inner cross-validation folds (i.e., 4-fold cross-validation AUPRC). The process is repeated for each ligand-classifier pair with an additional step of early stopping for NN and XGB to prevent overfitting. Once we identify the set of hyperparameters that yields the best validation performance, we train the model using these hyperparameters on all the training folds (the external cross-validation) and predict for the test fold. To avoid information leakage during the evaluation, all the data scaling procedures are run on the training partition only (both in the inner and outer cross-validation training), and the resulting outputs (e.g., normalization) are applied to the test data. The final reported performances for the base models are obtained by repeating this process for each of the five test folds and computing the mean AUPRC, AUC, and AUPRC-FI.

#### 1.4.4 Training and hyperparameter tuning for the stacked models

We train our stacked models with additional internal cross-validation within our nested-cross validation procedure (see Algorithm 19.8 Stacking with k-fold cross-validation in [38]). That is, when considering each of the five folds as the test fold, for each base model, we use the hyperparameter settings identified as above and train on all combinations of three folds to make out-of-fold predictions for all positions within all domains contained in the fourth fold. Then to choose hyperparameters for the second layer meta-model, we use the first layer predictions for the four folds (along with the original features for all architectures except M5L5F$f$) and use the inner four-fold cross-validation procedure described above. We then train the meta-model using these hyperparameters and data from all four folds to make predictions on the fifth test fold. Our fold stratification across all the ligands simultaneously enables us to use the same folds for the training of all the classifiers and combine out-of-fold predictions from different ligands free from data leakage.

#### 1.4.5 Model selection and final dSPRINT model training

The final predictive model for each ligand consists of two components: (1) trained base models for creating the first-layer predictions for the final stacked model and (2) a trained second-layer stacked model that gets as input the first-layer predictions and outputs the final per-position binding predictions. More specifically, we first identify the best architecture for each ligand by comparing the mean AUPRC of all the base models and the different ensembles in cross-validation testing. In particular, the ensemble architectures that lead to best performance are M1L5Ft for RNA, ion, and peptide; M5L1Ft for DNA; and M5L5Ft for small molecule. Then, for each base model included in the chosen architecture, we tune hyperparameters with the random search technique. Here, each of the five folds is used in turn as the validation set, and the mean AUPRC across folds is computed. The hyperparameter combination that has the highest mean AUPRC is then used for training in two settings. First, each of the base models is trained on the entire training set, resulting in final base models that will be used to make first-layer predictions for new data. Second, in order to perform hyperparameter tuning of the second layer, base models are trained using each set of four folds in order to make out-of-fold predictions for the fifth fold; these out-of-fold predictions will then be combined with the original domain features as input for the second layer for hyperparameter tuning. The hyperparameters for the second layer stacking model are tuned with the random search technique (using each of the five folds in turn as the validation set), and the hyperparameter combination with highest mean AUPRC across folds is used to train the stacked model on all five folds again using out-of-fold predictions for the base models). This results in a final trained stacked model that runs directly on new data predictions returned by the base models trained on the entire training set.

### 1.5 Whole-domain feature construction

The features used to train five classifiers to predict whether a domain binds DNA, RNA, small molecules, ion, or peptides consist of: (1) the per-domain-position outputs of the five selected dSPRINT models to predict whether individual positions within domains bind DNA, RNA, small molecule, ions, and peptides; and (2) a subset of the features described above. Because protein domains vary in length, we summarize the per-position properties into a fixed number of features. The specifics of our whole-domain features are described in more detail below.

**Per-domain-position binding score features.** For each ligand type, we obtain a binding score prediction between 0 and 1 for each position in each domain in our training set. We use the same five folds as when we trained classifiers to predict ligand-binding positions within domains. To avoid information leakage, for each test fold in turn and for each of the five ligand types, we train a classifier on the other four training folds to predict binding positions for the domains within it. The architecture of each classifier is chosen as the one that gave overall best performance in predicting ligand-binding positions. That is, we use M1L5Ft for RNA, ion, and peptide; M5L1Ft for DNA; and M5L5Ft for small molecule. In order to train these ensemble models, we train the base models that comprise them in four-fold cross-validation within the training folds and the base models predict binding for each training fold using the other three folds. This results in out-of-fold dSPRINT per-domain-position binding scores, obtained through models that were trained not using any information from the test fold.

Once models to predict binding positions for the five ligands types are trained, we compute per-ligand percentiles and $Z$-scores for each position's predicted scores with respect to all positions included within the folds in the training set. That is, our cutoffs for percentiles and our mean and standard deviation for $Z$-scores are calculated without the test fold predictions. For each domain, for each of the five ligand types, we encode as features the fraction of positions above the 25th, 50th, and 75th percentile of scores for that ligand type. We also include features corresponding to the fraction of positions above $Z$-scores of 0.5, 1, 2, and 3. Since domains with a higher proportion of ligand-binding positions are more likely to bind that ligand, these features are informative. However, they do not capture distances between binding positions. Multiple proximal predicted binding positions may give additional support for a ligand-binding domain as these binding positions are likely to be proximal in three dimensions and could bind a ligand together. We thus also included features to capture this spatial information. We define a sliding window of five positions across a domain as *interesting* if it has at least two positions with predicted scores for a specific ligand above the 75th percentile of scores. For each ligand type, the feature we encode is the number of interesting windows. As an additional feature for each ligand type, we also included the predicted binding scores' normalized Shannon entropy, using the calculation explained in Section 1.3.4; that is, the per-domain-position scores are divided by their norm and then these scores are treated as a probability distribution to compute the normalized entropy. The entropy captures the distribution of the within-domain scores, and a low entropy corresponds to domains where a small fraction of positions have scores above the chosen percentiles and $Z$-score thresholds. Finally, we also encode the maximum per-domain-position binding score as a feature since high scores may indicate a binding site. We note that for all domains, the percentile and $Z$-score features need to recomputed for each test fold, due to using different "out-of-fold" per-domain-position predictions; that is, the value for the same feature for the same domain differs based on what the test fold is. Altogether, this process yielded 50 features (10 features for each ligand type).

**Summary of original features.** For each domain, we add an additional five types of features: (1) length, (2) conservation, (3) genomic variation, (4) physicochemical properties, and (5) Pfam emission probabilities. For length, we include a feature for the domain length, as well as the mean protein length of those proteins that contain instances of the domain. For conservation, we add the mean PhastCons and PhyloP scores across all domain instances and all positions. We include both as features because they measure slightly different properties as described earlier. For genomic variation, we add features for the mean minor allele frequency (MAF) and BLOSUM score, again over all domain instances and positions. For physicochemical properties, we add the mean hydrophobicity index and the average number of positively charged amino acids. We also

use predicted structural properties, averaged across each domain instance and all positions including solvent accessibility, alpha-helix propensity, beta-sheet propensity, and turn propensity. Lastly, for the Pfam emission probabilities, we add the maximum value across positions for Cysteine, Histidine, Lysine, and Arginine, as well as the maximum emission emission probability across positions. We also include the number of positions that meet the Pfam conservation threshold defined earlier in Section 1.1.1. Altogether this group of 18 summary features is broad enough to cover many important aspects of our feature set but not so large as to create problems when moving to the smaller training set of domains (as opposed to positions) binding certain ligand types.

## 1.6 Algorithm Extensions

### 1.6.1 Iterative Group Stratification

The purpose of the original IterativeStratification algorithm [39] is to balance a multi-label dataset with respect to all of its labels when distributing examples into $k$ output groups. We added a group constraint to the original algorithm, where all examples within a group have to be kept together in the same fold. In our case, a group consists of all the positions of a particular domain, and a label is a particular ligand type when each ligand's positives and negatives are two separate labels. We thus have 10 labels total for our five ligand types. We would like to distribute the examples so that (1) for each ligand type, all the folds have similar numbers of positive examples involving that ligand type and similar numbers of negative examples involving that ligand type, and (2) all examples for the same domain are in one fold.

Before running the procedure, for each group, we determine which of the 10 labels are associated with it (i.e., there are examples from that domain that have that label). Only certain combinations of labels are observed across the groups. At each step of the algorithm, we choose which of the observed combination of labels we will handle next (i.e., for which we will assign all the groups that have that combination of labels into folds). We choose the combination of labels based upon prioritization rules that follow the logic of the original algorithm [39]: (1) prioritize groups that are associated with larger numbers of different labels as those groups can cause the most imbalance across the different labels, which can later be corrected by groups with a smaller number of labels, and (2) break ties by prioritizing "rare" labels (i.e., labels that have fewer examples left to distribute should be handled before the more common labels). That is, at each iteration, we choose the remaining combination of labels that involves the most number of labels, breaking ties by picking the one that has the label with fewest number of total examples that still need to be assigned to a fold. If that rarest label is found in multiple combination of labels, ties are broken randomly, as was done in the original algorithm. After deciding on the label combination, all the groups associated with it are handled one after the other in random order. In our case, the domains are handled in alphabetical order. Each group of examples is assigned to the fold which has the smallest number of examples for the rarest label.

### 1.6.2 Frequency-based pN/pS

For a particular position in a domain, we define the "pseudo-reference sequence" as the concatenation of the major allele codons (codons with the highest frequency) in each of the protein positions aligned to this protein domain position.

For each codon $i$ in the pseudo-reference sequence, we first compute the expected number of synonymous

($s_i$) and nonsynonymous ($n_i$) sites. This is done per the usual way of checking all nine possible mutations in a codon (i.e., three DNA bases, each of which can be mutated with uniform probabilities to any of the other three). This enables us to compute the total number of expected synonymous and nonsynonymous sites in a pseudo-reference sequence consisting of $r$ codons as $S = \sum_{i=1}^{r} s_i$ and $N = \sum_{i=1}^{r} n_i$, respectively.

We let $s_{d_i}$ and $n_{d_i}$ denote the number of synonymous and nonsynonymous changes observed for codon $i$. For each substitution $j$ occurring at reference codon $i$, where we assume the substitution arises from a single event mutation since we only model SNPs from ExAC, we determine the substitution effect (i.e., synonymous or nonsynonymous) and add its reported MAF to $s_{d_i}$ or $n_{d_i}$ respectively. This ensures that that each substitution effect is weighted by its population frequency. We next obtain the total number of synonymous and nonsynonymous differences by summing up over all codons: $S_d = \sum_{i=1}^{r} s_{d_i}$ and $N_d = \sum_{i=1}^{r} n_{d_i}$. We compute the proportion of synonymous ($q_S$) and nonsynonymous ($q_N$) substitutions as $q_S = \frac{S_d}{S}$ and $q_N = \frac{N_d}{N}$. Then pN/pS is determined from $q_N$ and $q_S$ as described in [40]:

$$p_N = -\frac{3}{4}\ln(1 - \frac{4q_N}{3}), \quad p_S = -\frac{3}{4}\ln(1 - \frac{4q_S}{3})$$

$$\text{pN/pS} = \frac{p_N}{p_S}$$

# References


[1] Zerbino, D. R., Achuthan, P., Akanni, W., Amode, M. R., Barrell, D., Bhai, J., Billis, K., Cummins, C., Gall, A., Girón, C. G., et al. (2017) Ensembl 2018. *Nucleic Acids Research,* **46**(D1), D754–D761.

[2] El-Gebali, S., Mistry, J., Bateman, A., Eddy, S. R., Luciani, A., Potter, S. C., Qureshi, M., Richardson, L. J., Salazar, G. A., Smart, A., Sonnhammer, E. L. L., et al. (2018) The Pfam protein families database in 2019. *Nucleic Acids Research,* **47**(D1), D427–D432.

[3] Eddy, S. R. (2011) Accelerated profile HMM searches. *PLoS Computational Biology,* **7**(10), e1002195.

[4] Steinegger, M., Meier, M., Mirdita, M., Voehringer, H., Haunsberger, S. J., and Soeding, J. (2019) HH-suite3 for fast remote homology detection and deep protein annotation. *BMC Bioinformatics,* **20**, 473.

[5] Berman, H. M., Westbrook, J., Feng, Z., Gilliland, G., Bhat, T. N., Weissig, H., Shindyalov, I. N., and Bourne, P. E. (2000) The protein data bank. *Nucleic Acids Research,* **28**(1), 235–242.

[6] Yang, J., Roy, A., and Zhang, Y. (2012) BioLiP: a semi-manually curated database for biologically relevant ligand–protein interactions. *Nucleic Acids Research,* **41**(D1), D1096–D1103.

[7] Siepel, A., Bejerano, G., Pedersen, J. S., Hinrichs, A. S., Hou, M., Rosenbloom, K., Clawson, H., Spieth, J., Hillier, L. W., Richards, S., et al. (2005) Evolutionarily conserved elements in vertebrate, insect, worm, and yeast genomes. *Genome Research,* **15**(8), 1034–1050.

[8] Pollard, K. S., Hubisz, M. J., Rosenbloom, K. R., and Siepel, A. (2010) Detection of nonneutral substitution rates on mammalian phylogenies. *Genome Research,* **20**(1), 110–121.

[9] Yang, Y., Heffernan, R., Paliwal, K., Lyons, J., Dehzangi, A., Sharma, A., Wang, J., Sattar, A., and Zhou, Y. (2017) Spider2: A package to predict secondary structure, accessible surface area, and main-chain torsional angles by deep neural networks. In *Prediction of Protein Secondary Structure* pp. 55–63 Springer.

[10] Lek, M., Karczewski, K. J., Minikel, E. V., Samocha, K. E., Banks, E., Fennell, T., O'Donnell-Luria, A. H., Ware, J. S., Hill, A. J., Cummings, B. B., et al. (2016) Analysis of protein-coding genetic variation in 60,706 humans. *Nature,* **536**(7616), 285.

[11] Sim, N.-L., Kumar, P., Hu, J., Henikoff, S., Schneider, G., and Ng, P. C. (2012) SIFT web server: predicting effects of amino acid substitutions on proteins. *Nucleic Acids Research,* **40**(W1), W452–W457.

[12] Adzhubei, I. A., Schmidt, S., Peshkin, L., Ramensky, V. E., Gerasimova, A., Bork, P., Kondrashov, A. S., and Sunyaev, S. R. (2010) A method and server for predicting damaging missense mutations. *Nature Methods,* **7**(4), 248.

[13] Landrum, M. J., Lee, J. M., Benson, M., Brown, G., Chao, C., Chitipiralla, S., Gu, B., Hart, J., Hoffman, D., Hoover, J., et al. (2015) ClinVar: public archive of interpretations of clinically relevant variants. *Nucleic Acids Research,* **44**(D1), D862–D868.

[14] McKenna, A., Hanna, M., Banks, E., Sivachenko, A., Cibulskis, K., Kernytsky, A., Garimella, K., Altshuler, D., Gabriel, S., Daly, M., et al. (2010) The Genome Analysis Toolkit: a MapReduce framework for analyzing next-generation DNA sequencing data. *Genome Research,* **20**(9), 1297–1303.

[15] DePristo, M. A., Banks, E., Poplin, R., Garimella, K. V., Maguire, J. R., Hartl, C., Philippakis, A. A., Del Angel, G., Rivas, M. A., Hanna, M., et al. (2011) A framework for variation discovery and genotyping using next-generation DNA sequencing data. *Nature Genetics,* **43**(5), 491.

[16] Capra, J. A. and Singh, M. (2007) Predicting functionally important residues from sequence conservation. *Bioinformatics,* **23**(15), 1875–1882.

[17] Cooper, G. M., Hausman, R. E., and Hausman, R. E. The cell: a molecular approach Vol. 2, p. 51 ASM press Washington, DC (2000).

[18] Nelson, D. L., Lehninger, A. L., and Cox, M. M. (2008) Lehninger principles of biochemistry, Macmillan, .

[19] Kyte, J. and Doolittle, R. F. (1982) A simple method for displaying the hydropathic character of a protein. *Journal of Molecular Biology,* **157**(1), 105–132.

[20] Zamyatnin, A. (1972) Protein volume in solution. *Progress in Biophysics and Molecular Biology,* **24**, 107–123.

[21] Valdar, W. S. (2002) Scoring residue conservation. *Proteins: Structure, Function, and Bioinformatics,* **48**(2), 227–241.

[22] McDonald, I. K. and Thornton, J. M. (1994) Satisfying hydrogen bonding potential in proteins. *Journal of Molecular Biology,* **238**(5), 777–793.

[23] Chou, P. Y. and Fasman, G. D. (1978) Empirical predictions of protein conformation. *Annual Review of Biochemistry,* **47**(1), 251–276.

[24] Hamelryck, T. (2005) An amino acid has two sides: a new 2D measure provides a different view of solvent exposure. *Proteins: Structure, Function, and Bioinformatics,* **59**(1), 38–48.

[25] Heffernan, R., Dehzangi, A., Lyons, J., Paliwal, K., Sharma, A., Wang, J., Sattar, A., Zhou, Y., and Yang, Y. (2015) Highly accurate sequence-based prediction of half-sphere exposures of amino acid residues in proteins. *Bioinformatics,* **32**(6), 843–849.

[26] Faraggi, E., Yang, Y., Zhang, S., and Zhou, Y. (2009) Predicting continuous local structure and the effect of its substitution for secondary structure in fragment-free protein structure prediction. *Structure,* **17**(11), 1515–1527.

[27] Lyons, J., Dehzangi, A., Heffernan, R., Sharma, A., Paliwal, K., Sattar, A., Zhou, Y., and Yang, Y. (2014) Predicting backbone C$\alpha$ angles and dihedrals from protein sequences by stacked sparse auto-encoder deep neural network. *Journal of Computational Chemistry,* **35**(28), 2040–2046.

[28] Miller, M. L., Reznik, E., Gauthier, N. P., Aksoy, B. A., Korkut, A., Gao, J., Ciriello, G., Schultz, N., and Sander, C. (2015) Pan-cancer analysis of mutation hotspots in protein domains. *Cell Systems,* **1**(3), 197–209.

[29] McDonald, J. H. and Kreitman, M. (1991) Adaptive protein evolution at the Adh locus in Drosophila. *Nature,* **351**(6328), 652.

[30] Yang, Z. (2007) PAML 4: phylogenetic analysis by maximum likelihood. *Molecular Biology and Evolution,* **24**(8), 1586–1591.

[31] Capra, J. A. and Singh, M. (2008) Characterization and prediction of residues determining protein functional specificity. *Bioinformatics,* **24**(13), 1473–1480.

[32] Sander, C. and Schneider, R. (1991) Database of homology-derived protein structures and the structural meaning of sequence alignment. *Proteins: Structure, Function, and Bioinformatics,* **9**(1), 56–68.

[33] Pedregosa, F., Varoquaux, G., Gramfort, A., Michel, V., Thirion, B., Grisel, O., Blondel, M., Prettenhofer, P., Weiss, R., Dubourg, V., Vanderplas, J., Passos, A., Cournapeau, D., Brucher, M., Perrot, M., and Duchesnay, E. (2011) Scikit-learn: Machine Learning in Python. *Journal of Machine Learning Research,* **12**, 2825–2830.

[34] Chen, T. and Guestrin, C. (2016) Xgboost: A scalable tree boosting system. In *Proceedings of the 22nd ACM SIGKDD International Conference on Knowledge Discovery and Data Mining* ACM pp. 785–794.

[35] Paszke, A., Gross, S., Chintala, S., Chanan, G., Yang, E., DeVito, Z., Lin, Z., Desmaison, A., Antiga, L., and Lerer, A. (2017) Automatic differentiation in PyTorch. In *NIPS-W*.

[36] Bergstra, J. and Bengio, Y. (2012) Random search for hyper-parameter optimization. *Journal of Machine Learning Research,* **13**(Feb), 281–305.

[37] Bengio, Y. (2012) Practical recommendations for gradient-based training of deep architectures. In *Neural networks: Tricks of the trade* pp. 437–478 Springer.

[38] Aggarwal, C. C. Data classification: algorithms and applications pp. 498–501 CRC press (2014).

[39] Sechidis, K., Tsoumakas, G., and Vlahavas, I. (2011) On the stratification of multi-label data. In *Joint European Conference on Machine Learning and Knowledge Discovery in Databases* Springer pp. 145–158.

[40] Nei, M. and Gojobori, T. (1986) Simple methods for estimating the numbers of synonymous and nonsynonymous nucleotide substitutions. *Molecular Biology and Evolution,* **3**(5), 418–426.

[41] Peled, S., Leiderman, O., Charar, R., Efroni, G., Shav-Tal, Y., and Ofran, Y. (2016) De-novo protein function prediction using DNA binding and RNA binding proteins as a test case. *Nature Communications,* **7**, 13424.

[42] Yan, J. and Kurgan, L. (2017) DRNApred, fast sequence-based method that accurately predicts and discriminates DNA-and RNA-binding residues. *Nucleic Acids Research,* **45**(10), e84–e84.

[43] Zhang, J. and Kurgan, L. (2019) SCRIBER: accurate and partner type-specific prediction of protein-binding residues from proteins sequences. *Bioinformatics,* **35**(14), i343–i353.

[44] Porollo, A. and Meller, J. (2007) Prediction-based fingerprints of protein–protein interactions. *Proteins: Structure, Function, and Bioinformatics,* **66**(3), 630–645.

[45] Cao, X., Hu, X., Zhang, X., Gao, S., Ding, C., Feng, Y., and Bao, W. (2017) Identification of metal ion binding sites based on amino acid sequences. *PloS One,* **12**(8), e0183756.

[46] Liu, G.-H., Shen, H.-B., and Yu, D.-J. (2016) Prediction of protein–protein interaction sites with machine-learning-based data-cleaning and post-filtering procedures. *The Journal of Membrane Biology,* **249**(1), 141–153.

[47] Zhou, J., Lu, Q., Xu, R., Gui, L., and Wang, H. (2016) Cnnsite: Prediction of dna-binding residues in proteins using convolutional neural network with sequence features. In *2016 IEEE International Conference on Bioinformatics and Biomedicine (BIBM)* IEEE pp. 78–85.

[48] Wei, Z.-S., Han, K., Yang, J.-Y., Shen, H.-B., and Yu, D.-J. (2016) Protein–protein interaction sites prediction by ensembling SVM and sample-weighted random forests. *Neurocomputing,* **193**, 201–212.

[49] Jia, J., Liu, Z., Xiao, X., Liu, B., and Chou, K.-C. (2016) iPPBS-Opt: a sequence-based ensemble classifier for identifying protein-protein binding sites by optimizing imbalanced training datasets. *Molecules,* **21**(1), 95.

[50] Taherzadeh, G., Yang, Y., Zhang, T., Liew, A. W.-C., and Zhou, Y. (2016) Sequence-based prediction of protein–peptide binding sites using support vector machine. *Journal of Computational Chemistry,* **37**(13), 1223–1229.

[51] Hu, X., Dong, Q., Yang, J., and Zhang, Y. (2016) Recognizing metal and acid radical ion-binding sites by integrating ab initio modeling with template-based transferals. *Bioinformatics,* **32**(21), 3260–3269.

[52] Miao, Z. and Westhof, E. (2015) Prediction of nucleic acid binding probability in proteins: a neighboring residue network based score. *Nucleic Acids Research,* **43**(11), 5340–5351.

[53] Xiong, D., Zeng, J., and Gong, H. (2015) RBRIdent: An algorithm for improved identification of RNA-binding residues in proteins from primary sequences. *Proteins: Structure, Function, and Bioinformatics,* **83**(6), 1068–1077.

[54] Dhole, K., Singh, G., Pai, P. P., and Mondal, S. (2014) Sequence-based prediction of protein–protein interaction sites with L1-logreg classifier. *Journal of Theoretical Biology,* **348**, 47–54.

[55] Walia, R. R., Xue, L. C., Wilkins, K., El-Manzalawy, Y., Dobbs, D., and Honavar, V. (2014) RNABindRPlus: a predictor that combines machine learning and sequence homology-based methods to improve the reliability of predicted RNA-binding residues in proteins. *PloS One,* **9**(5), e97725.

[56] Jordan, R. A., Yasser, E.-M., Dobbs, D., and Honavar, V. (2012) Predicting protein-protein interface residues using local surface structural similarity. *BMC Bioinformatics,* **13**(1), 1–14.

[57] Wang, L., Huang, C., Yang, M. Q., and Yang, J. Y. (2010) BindN+ for accurate prediction of DNA and RNA-binding residues from protein sequence features. *BMC Systems Biology,* **4**(1), 1–9.

[58] Murakami, Y. and Mizuguchi, K. (2010) Applying the Naïve Bayes classifier with kernel density estimation to the prediction of protein–protein interaction sites. *Bioinformatics,* **26**(15), 1841–1848.

[59] Terribilini, M., Sander, J. D., Lee, J.-H., Zaback, P., Jernigan, R. L., Honavar, V., and Dobbs, D. (2007) RNABindR: a server for analyzing and predicting RNA-binding sites in proteins. *Nucleic Acids Research,* **35**(suppl_2), W578–W584.

[60] Kumar, M., Gromiha, M. M., and Raghava, G. P. (2007) Identification of DNA-binding proteins using support vector machines and evolutionary profiles. *BMC Bioinformatics,* **8**(1), 463.

[61] Ahmad, S. and Sarai, A. (2005) PSSM-based prediction of DNA binding sites in proteins. *BMC Bioinformatics,* **6**(1), 1–6.

# 2 Supplementary figures

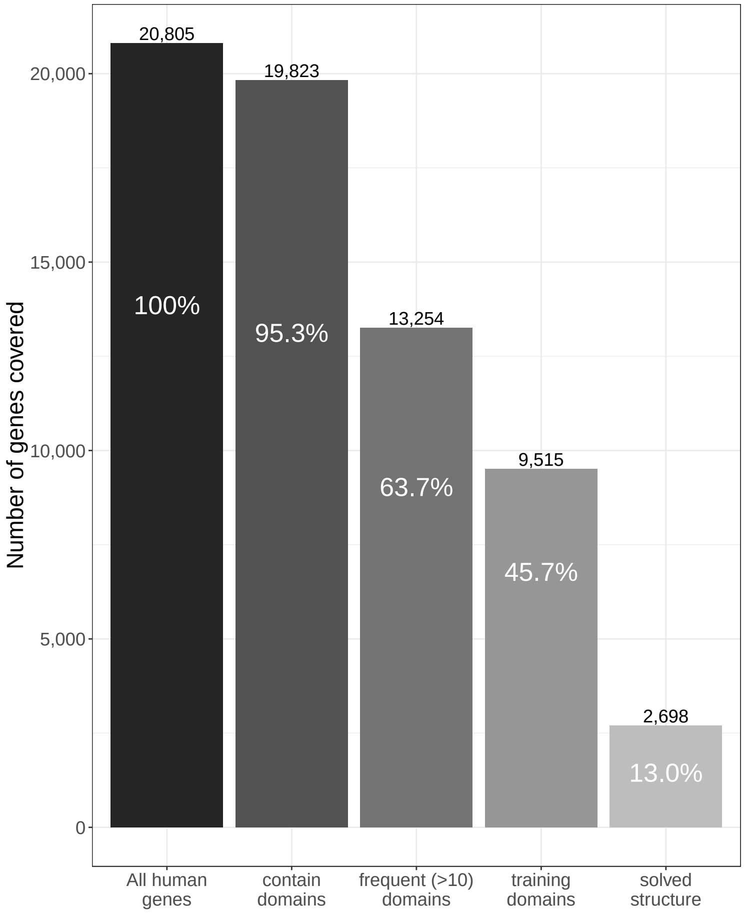


**Figure S1: Domain-centric approach coverage of human genes.** Nearly all the human genes contain at least one domain instance (>95%) and most genes contain domains that have more than 10 instances in the human genome (~2/3 of the genes). Our training set consist of domains that have at least 10 instances in the human genome and that have InteracDome structurally-derived binding scores. Nearly half of human genes contain an instance of at least one domain that is in our training set. In contrast, only ~13% of human genes are present in some co-complex crystal structure, as determined by counting the number of human genes that are found in complex with a ligand in Biolip [6].

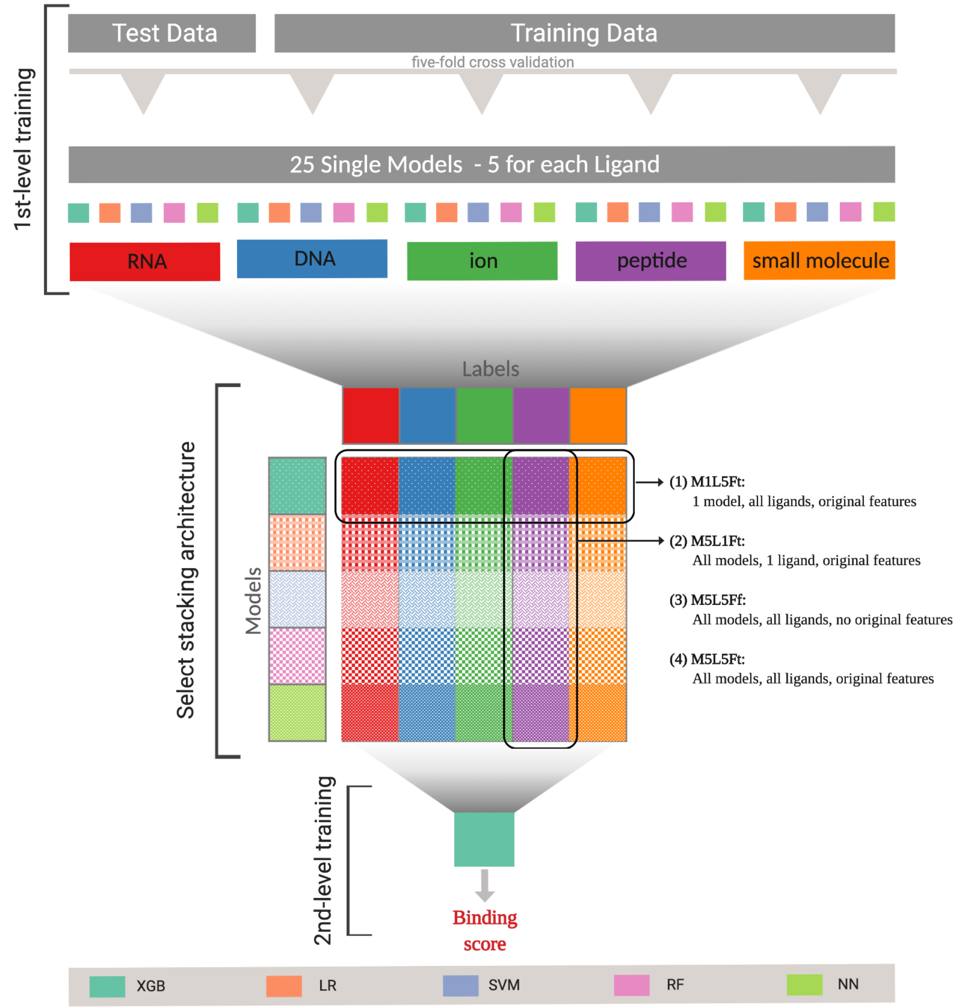


**Figure S2: Stacking architecture.** Block diagram of the proposed ensemble system. First, the data is split into testing and training sets, and five base models (XGB, LR, SVM, RF, and NN) are trained for each ligand in 5-fold cross validation. This results in 25 base models that are used in four combinations for the second-level training, as illustrated using the colorful grid. The stacking architectures combine models (shown vertically to the left of the grid in patterns) and the outputs of the trained base models for ligands (shown horizontally above the grid in solid colors). We consider four different ensembles: (1) M1L5Ft, highlighted in the figure as the first row in the grid, consists of XGB trained on the outputs of the base model XGB trained for all five ligands and all the original features; (2) M5L1Ft, corresponding to columns in the grid and highlighted in the figure for peptide, consists of XGB trained on the outputs of all five trained base models for a particular ligand and all the original features; (3) M5L5Ff consists of LR trained on the outputs of all 25 trained base models (i.e,. the full grid) and without the original features; and (4) M5L5Ft consists of XGB trained on the outputs of all 25 trained base models and all the original features.

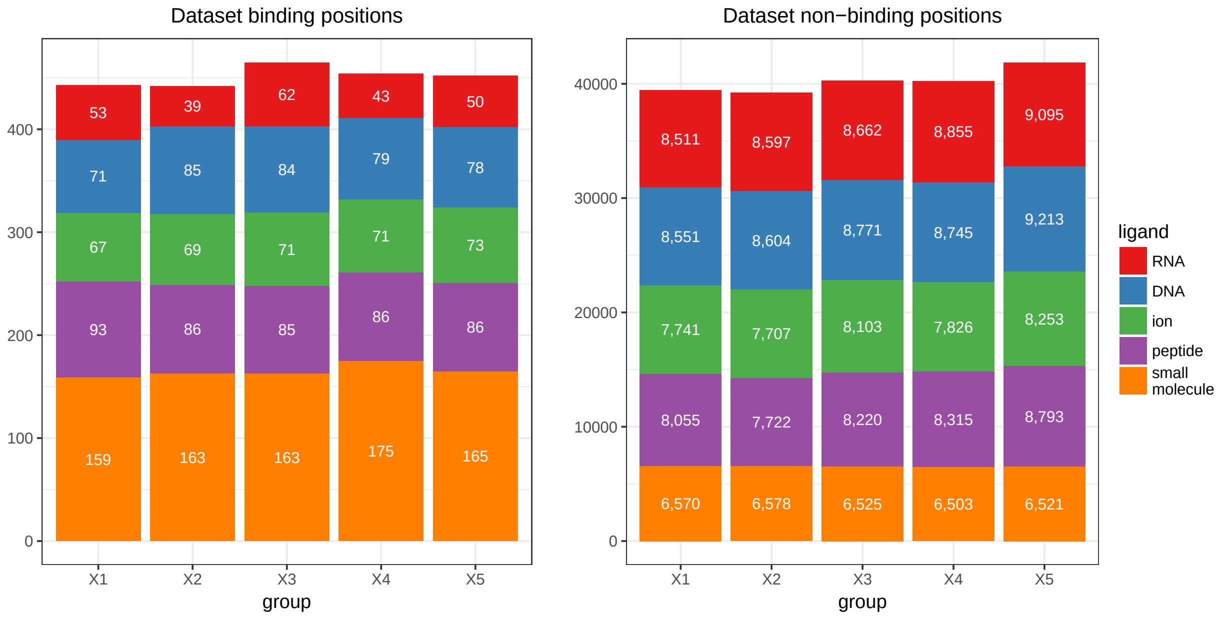


**Figure S3: Balanced number of examples in each cross-validation fold.** The *IterativeGroupStratification* algorithm was run on the entire set of binding and non-binding positions across all ligand types in order to generate five folds that are similar to each other with respect to the number of binding and non-binding positions for each ligand. The groups were stratified with respect to all the ligands at the same time, with an added constraint of keeping all the positions of the same domain together. On the left, we give the number of binding positions in each fold for each ligand. On the right, we give the number of non-binding positions in each fold for each ligand.

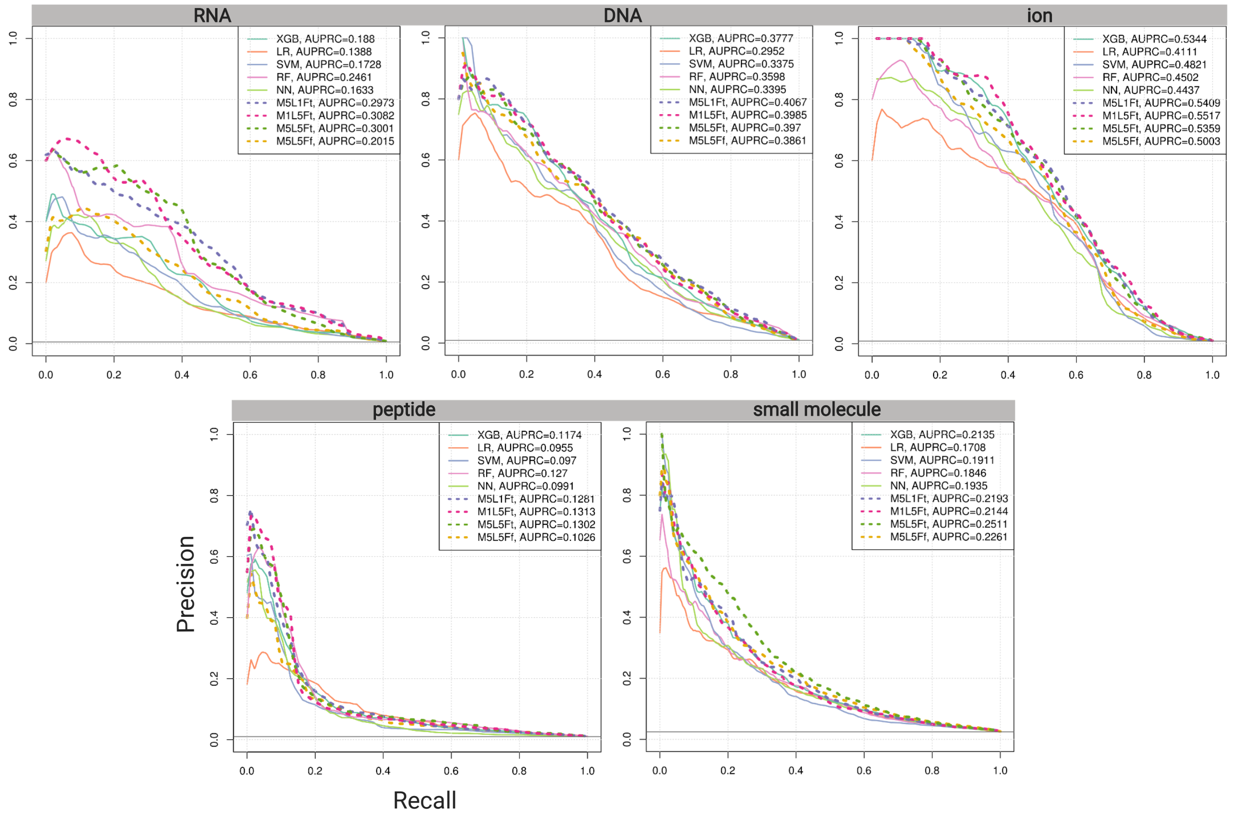


**Figure S4: PR curves in cross-validation experiments.** For each of the five ligands, we give PR curves and AUPRCs for our five base models and four stacking models. Each curve shows the mean precision and mean recall averaged across the five folds.

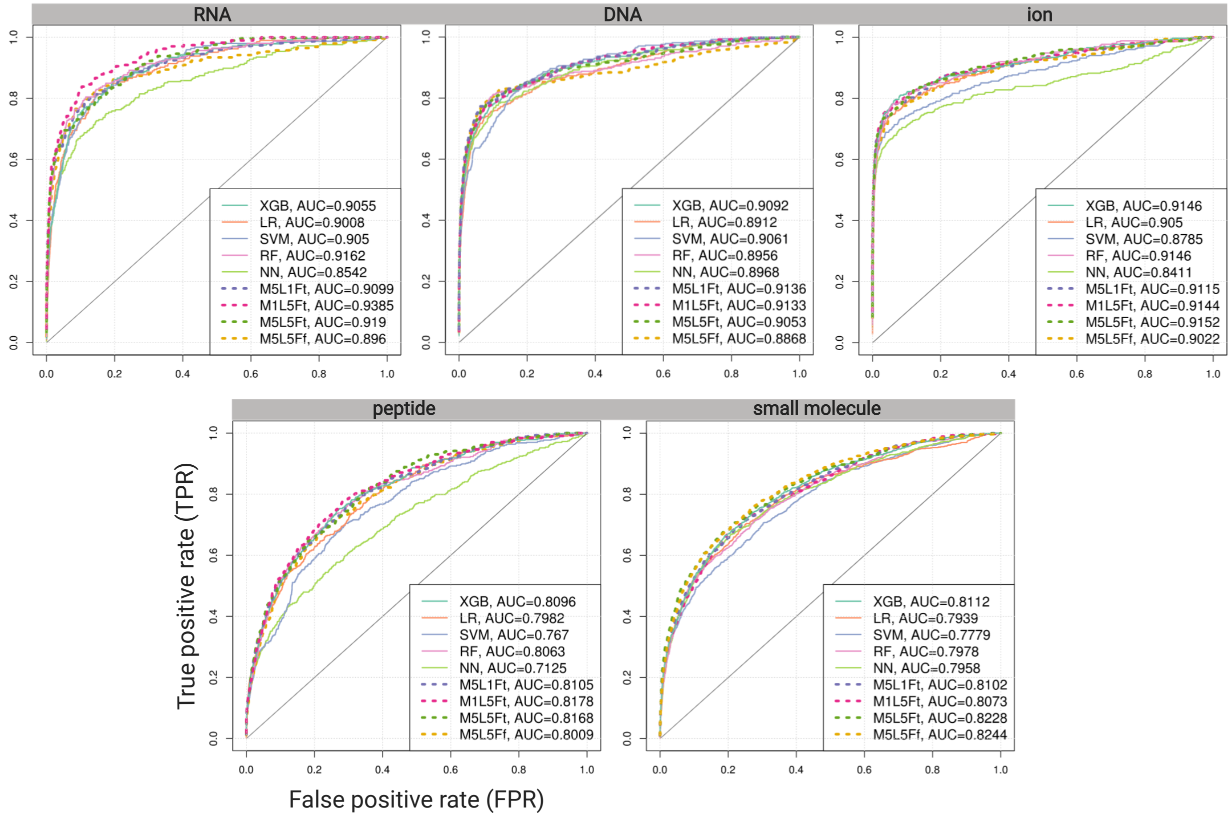


**Figure S5: ROC curves in cross-validation experiments.** For each of the five ligands, we give ROC curves and AUCs for our five base models and four stacking models. Each curve shows the mean true positive rate (TPR) and mean false positive rate (FPR) averaged across the five folds.

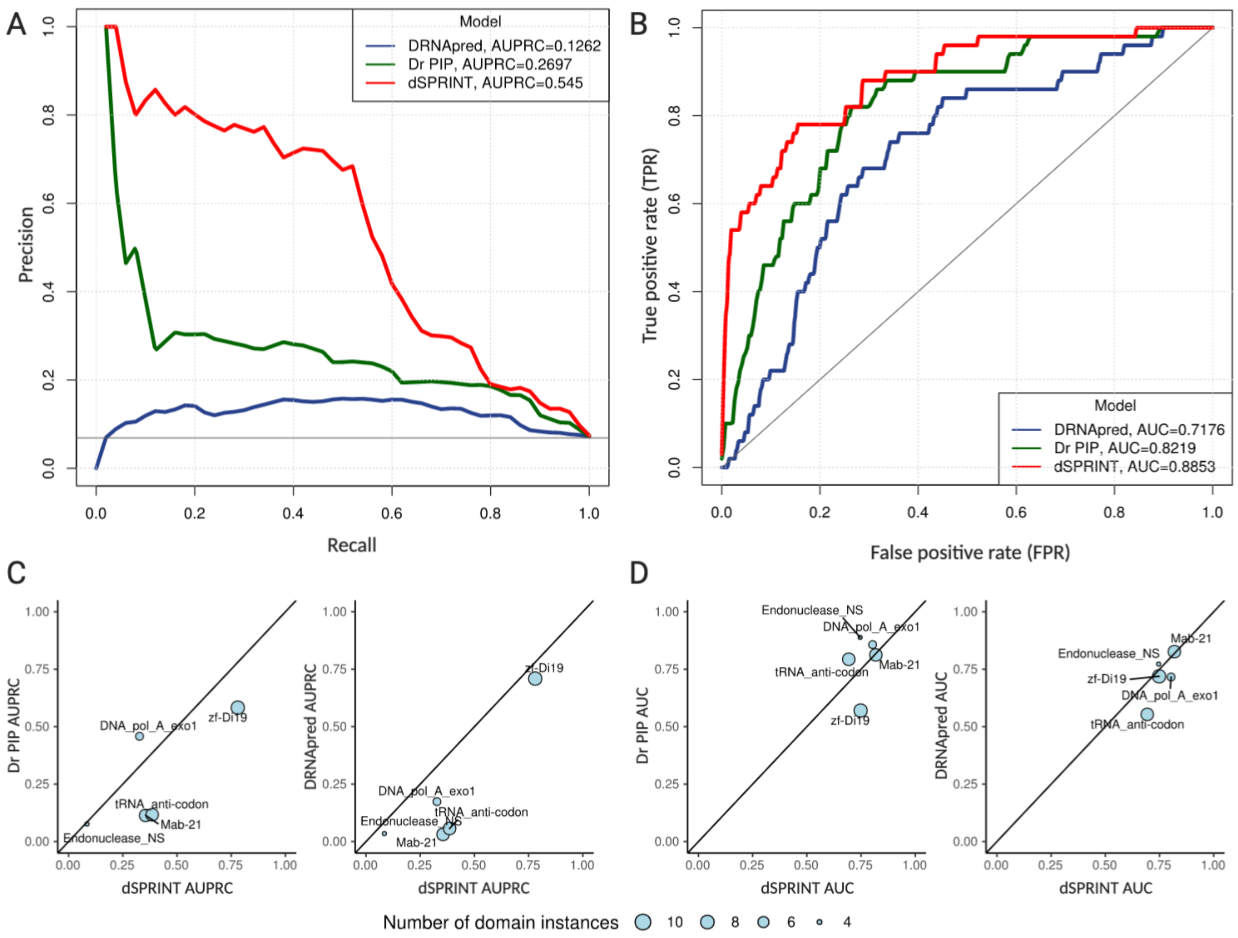


**Figure S6: Performance of dSPRINT as compared to aggregating predictions from methods for predicting DNA-binding sites within protein sequences** We compare dSPRINT to aggregating by domain position the amino acid-level predictions of Dr PIP [41] and DRNApred [42]. (**A**) PR curves and AUPRCs. (**B**) ROC curves and AUCs. (**C**) Per-domain AUPRCs comparing dSPRINT with aggregating Dr PIP (left) and DRNApred (right). (**D**) Per-domain AUCs comparing dSPRINT with Dr PIP (left) and DRNApred (right). For both (C) and (D), the size of the dots corresponds to the number of instances.

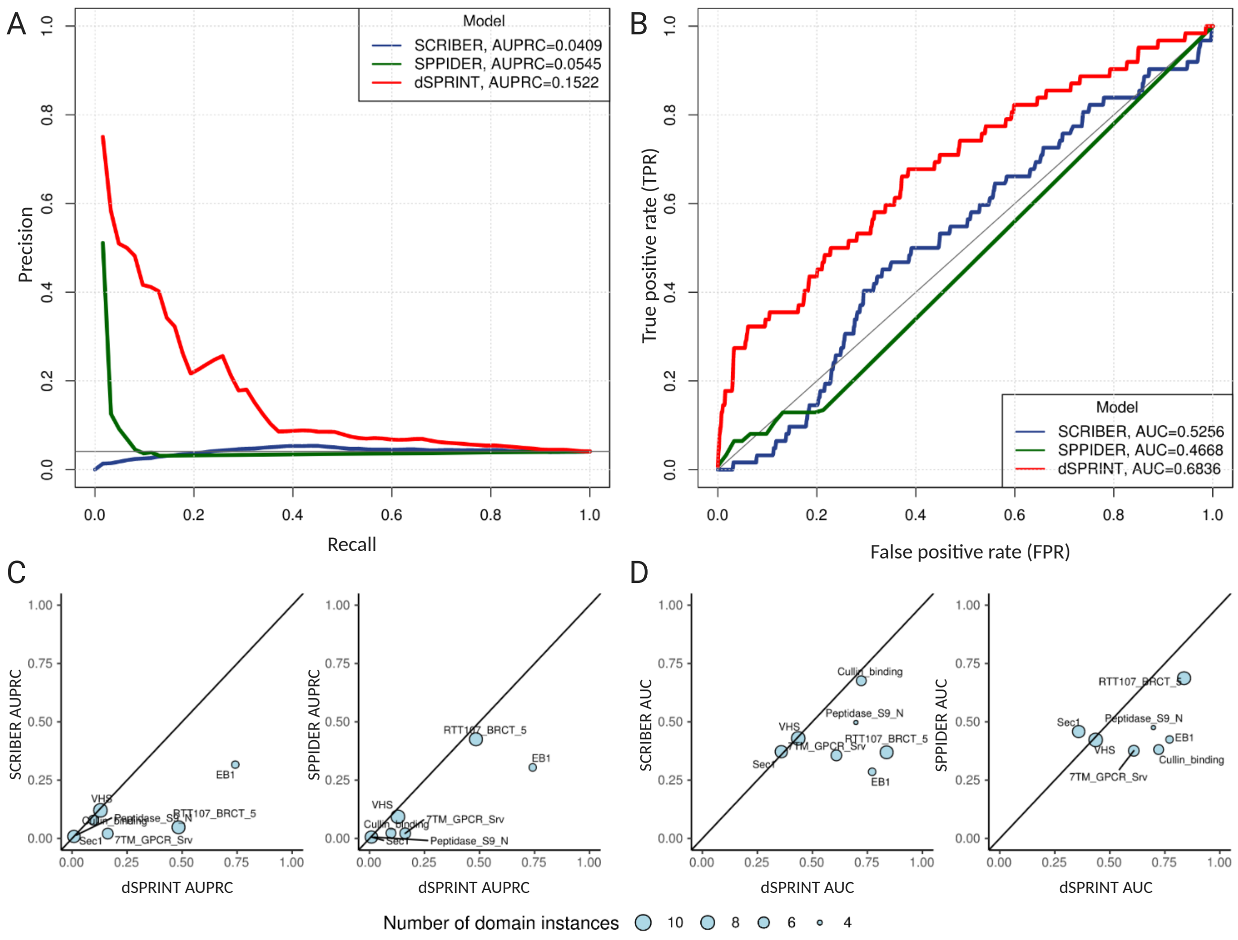


**Figure S7: Performance of dSPRINT as compared to aggregating predictions from methods for predicting peptide-binding sites within protein sequences.** We compare dSPRINT to aggregating by domain position the amino acid-level predictions of SCRIBER [43] and SPPIDER [44]. (**A**) PR curves and AUPRCs. (**B**) ROC curves and AUCs. (**C**) Per-domain AUPRCs comparing dSPRINT with aggregating SCRIBER (left) and SPPIDER (right). (**D**) Per-domain AUCs comparing dSPRINT with SCRIBER (left) and SPPIDER (right). For both (C) and (D), the size of the dots corresponds to the number of instances. We note that SPPIDER predicted 80% of the positions with a binding score of 0.

# 3 Supplementary tables

| Sup. section | Feature group | Feature subgroup | # (w#) | Total |
|---|---|---|---|---|
| 1.3.1 | Conservation | DNA-level | 86 (3) | 115 |
| | | Amino acid-level | 7 (1) | |
| | | Domain-level | 22 (1) | |
| 1.3.2 | Physiochemical | Identity | 42 | 66 |
| | | Charge | 4 (1) | |
| | | Functional group | 5 | |
| | | Hydrophobicity index | 3 (1) | |
| | | Volume | 4 (1) | |
| | | Hydrogen bonds | 2 (2) | |
| | | Secondary structure propensity | 6 (3) | |
| 1.3.3 | Structurally-predicted | Surface accessibility | 2 (2) | 31 |
| | | Contact number | 4 (2) | |
| | | Half-sphere exposure | 8 (4) | |
| | | Secondary structure predictions | 9 (3) | |
| | | Backbone angles | 8 (4) | |
| 1.3.4 | Population variant-based | Variant frequencies | 33 (1) | 199 |
| | | Variant counts | 6 | |
| | | Purifying selection | 3 | |
| | | Non-synonymous variant distribution | 1 | |
| | | Identity of variant amino acids | 84 | |
| | | Substitution matrices | 10 (2) | |
| | | Variant effect predictors | 14 (3) | |
| | | Variant physiochemical transitions | 48 (4) | |
| 1.3.5 | Selection-based | dN/dS inspired | 3 (1) | 13 |
| | | Ortholog-paralog conservation ratio | 10 (1) | |
| 1.3.6 | Location and length | Domain-context | 5 | 9 |
| | | Protein-context | 4 | |
| 1.3.7 | Windowed features | Conservation | 40 | 320 |
| | | Physiochemical | 64 | |
| | | Structurally-predicted | 120 | |
| | | Population variant-based | 80 | |
| | | Selection-based | 16 | |

**Table S1: Summary of features.** An overview of the features used to train our base models, as described in Section 1.3. For each feature type, we list the number of encoded features and in parenthesis give the number of them that were used to compute windowed features. Note that for each regular feature used as a windowed feature (denoted by (w) in the text), we compute eight windowed features (i.e., for each of four window sizes, we compute the mean and standard deviation of the feature across the window).

| Algorithm | Tuned parameters | Initial range to sample from |
|---|---|---|
| XGB | n_estimators - Number of boosted trees to fit. | Determined with early stopping. |
| | max_depth - Maximum tree depth for base learners. | [1, 100] |
| | learning_rate - Boosting learning rate. | [1e−4, 1e−0.5] |
| | gamma - Minimum loss reduction required to make a further partition on a leaf node of the tree. | [1e−3, 1] |
| | min_child_weight - Minimum sum of instance weight needed in a child. | [0, 20] |
| | colsample_bytree - Subsample ratio of columns when constructing each tree. | [0.1, 1] |
| | scale_pos_weight - Balancing of positive and negative weights. | {1, balanced, 0.1} |
| LR | C - Inverse of regularization strength | [1e−3, 1] |
| | class_weight - Weights associated with classes. | {None, balanced, 0 : 10, 1 : 1} |
| SVM | n_estimators - Number of boosted trees to fit. | Determined with early stopping. |
| | kernel - the kernel type to be used in the algorithm | rbf* |
| | C - Penalty parameter C of the error term. | [1e−4, 1e1] |
| | class_weight - Set the parameter C of each class | {None, balanced, 0 : 10, 1 : 1} |
| RF | n_estimators - The number of trees in the forest. | [10, 1500] |
| | max_depth - The maximum depth of the tree. | [2, 100] |
| | min_samples_split - The minimum number of samples required to split an internal node. | [2, 50] |
| | min_samples_leaf - The minimum number of samples required to be at a leaf node. | [1, 50] |
| | class_weight - Weights associated with classes. | balanced* |
| NN | epoch_count – The number times the dataset is passed forward and backward through the network. | Determined with early stopping. |
| | batch_size – The number of training examples in one forward/backward pass. | [30, 300] |
| | Hidden_1 – the number of neurons in the 1st hidden layer. | [200, 1000] |
| | Hidden_2 – the number of neurons in the 2nd hidden layer. | [350, 1000] |
| | weight – A manual rescaling weight given to the loss of each batch element. | {None, balanced, 0.1} |
| | learning_rate – Adam optimizer learning rate, the proportion that weights are updated. | ion: [1e−4, 1e−2], peptide: [1e−5, 1e−3], RNA, DNA, small molecule: [1e−6, 1e−4], |
| | beta – Adam optimizer exponential decay rate for the first moment estimate. | [0.8, 0.99] |
| | weight_decay – Adam optimizer magnitude in which the weights influence is decremented after each update. | [1e−25, 1e−5] |

**Table S2: Model Hyperparameters.** For each classifier type, we give a summary of the hyperparameters and the range of values used in the tuning process. The two hyperparameters that are starred were fixed to the given values.

| Tool | Reference | Ligand | Comments |
|---|---|---|---|
| **SCRIBER (2019)** | **[43]** | **PPI** | **http://biomine.cs.vcu.edu/servers/SCRIBER/** |
| **DRNApred (2017)** | **[42]** | **DNA, RNA** | **http://biomine.cs.vcu.edu/servers/DRNApred** |
| Cao et al. (2017) | [45] | ion | Broken link: http://60.31.198.140:8081/metal/HomePage/HomePage.html |
| **Dr PIP (2016)** | **[41]** | **DNA, RNA** | **http://ofranservices.biu.ac.il/site/services/dr_pip/index.html** |
| PPIs (2016) | [46] | PPI | Does not supply per-position predictions: http://csbio.njust.edu.cn/bioinf/PPIS/ |
| CNNsite (2016) | [47] | DNA | Broken link: http://hlt.hitsz.edu.cn:8080/CNNsite/ |
| SSWRF (2016) | [48] | PPI | Service no longer provided: http://csbio.njust.edu.cn/bioinf/SSWRF |
| iPPBS-Opt (2016) | [49] | PPI | Returned a blank prediction for all the inputs: http://www.jci-bioinfo.cn/iPPBS-Opt |
| SPRINT (2016) | [50] | PPI | Broken link: : https://sparks-lab.org/server/SPRINT/ |
| IonSeq (2016) | [51] | ion | Broken link:: http://zhanglab.ccmb.med.umich.edu/IonCom |
| Rbscore (2015) | [52] | DNA, RNA | Broken link: http://ahsoka.u-strasbg.fr/rbscore/ |
| RBRIdent (2015) | [53] | RNA | Broken link: http://166.111.152.91/RBRIdent |
| LORIS (2014) | [54] | PPI | Tool website is working: https://sites.google.com/site/sukantamondal/software But running a sequence requires submission to another website that is not available: http://lee.kias.re.kr/ newton/sann/ |
| RNABindRPlus (2014) | [55] | RNA | Broken link to tool datasets: http://einstein.cs.iastate.edu/RNABindRPlus/datasets.html |
| PrISE (2012) | [56] | PPI | Broken link: http://prise.cs.iastate.edu/ |
| BinN+ (2010) | [57] | DNA, RNA | Broken link: http://bioinfo.ggc.org/bindn+/ |
| PSIVER (2010) | [58] | PPI | Broken link: http://tardis.nibio.go.jp/PSIVER/ |
| RNABindR (2007) | [59] | RNA | Broken link: http://pridb.gdcb.iastate.edu/rnabindr.html |
| **SSPIDER (2007)** | **[44]** | **PPI** | **http://sppider.cchmc.org/** |
| DNAbinder (2007) | [60] | DNA | Does not supply per-position predictions: https://webs.iiitd.edu.in/raghava/dnabinder/ |
| DNABIND (2006) | [60] | DNA | No website/source code available |
| DBS-PSSM (2005) | [61] | DNA | Broken link: http://ccbb.jnu.ac.in/shandar/servers/dbs-pssm/ |

**Table S3: Methods considered for the comparative analysis.** We examined methods that predict sites within protein sequences that bind specific ligand types in order to create baseline methods for predicting binding positions within domains based on aggregating their results by domain position. Methods that we used in the comparative analysis described in the main manuscript are highlighted in bold, and for the other methods, the Comments column lists the reason for not including them in the analysis.

| Ligand | # of highest scoring predictions per domain | # of domains with at least one correct prediction | % of domains with at least one correct prediction |
|---|---|---|---|
| RNA | 1 | 13 | 62% |
| | 3 | 16 | 76% |
| | 5 | 18 | 86% |
| DNA | 1 | 22 | 67% |
| | 3 | 27 | 82% |
| | 5 | 29 | 88% |
| ion | 1 | 47 | 52% |
| | 3 | 60 | 66% |
| | 5 | 64 | 70% |
| peptide | 1 | 29 | 40% |
| | 3 | 44 | 61% |
| | 5 | 48 | 67% |
| small molecule | 1 | 72 | 55% |
| | 3 | 96 | 73% |
| | 5 | 104 | 79% |

**Table S4: dSPRINT's performance when considering the top-scoring 1, 3 or 5 positions within each domain.** For each ligand, and each number of top-scoring positions considered per domain, we give the number and percent of domains for which at least one of these predictions is actually a binding position. When considering only the highest scoring position for every protein domain, dSPRINT is correct for more than 40% of domains for each of the ligand types. When considering the five top predictions, at least one is correct for more than 67% of domains for each of the ligand types.

| DUF | # | Genes symbols/ids | Prediction score | Estimated precision |
|---|---|---|---|---|
| **RNA** | | | | |
| DUF3697 | 2 | SUBAP2, UBAP2L | 0.622146 | 0.33 |
| DUF106 | 2 | EMC3, TMCO1 | 0.582572 | 0.33 |
| DUF4535 | 2 | STMP1 | 0.540265 | 0.33 |
| DUF1897 | 1 | FUBP1 | 0.533172 | 0.33 |
| DUF3385 | 1 | MTOR | 0.502961 | 0.33 |
| DUF2363 | 1 | CNOT11 | 0.493661 | 0.33 |
| **DNA** | | | | |
| DUF4599 | 11 | SPATA31A1, SPATA31A6, SPATA31D1, SPATA31E1, C5orf60, FAM205A | 0.763326 | 0.84 |
| DUF1087 | 3 | CHD3, CHD4, CHD5 | 0.758519 | 0.84 |
| DUF1154 | 2 | PLCB1, PLCB4 | 0.758519 | 0.84 |
| DUF1220 | 307 | NBPF1, NBPF3, NBPF4, NBPF6, NBPF20, PDE4DIP | 0.73372 | 0.84 |
| DUF4519 | 1 | SMCO4 | 0.721624 | 0.83 |
| DUF1713 | 1 | AURKAIP1 | 0.696677 | 0.83 |
| DUF4629 | 1 | C2orf78 | 0.692026 | 0.83 |
| DUF1897 | 1 | FUBP1 | 0.592497 | 0.81 |
| DUF4074 | 3 | HOXA3, HOXB3, HOXD3 | 0.582876 | 0.80 |
| DUF2371 | 3 | TMEM200A, TMEM200B, TMEM200C | 0.503507 | 0.75 |
| **ion** | | | | |
| DUF4801 | 1 | MGA | 0.892697 | 0.80 |
| DUF4187 | 1 | GPATCH11 | 0.880241 | 0.80 |
| DUF504 | 3 | ENSG00000182909 | 0.839195 | 0.80 |
| DUF2043 | 1 | UVSSA | 0.819904 | 0.80 |
| DUF902 | 2 | CREBBP, EP300 | 0.813957 | 0.80 |
| DUF384 | 2 | HGH1 | 0.797212 | 0.80 |
| DUF1897 | 1 | FUBP1 | 0.774272 | 0.80 |
| DUF4339 | 1 | DNAJC13 | 0.757904 | 0.80 |
| DUF971 | 3 | BBOX1, TMLHE | 0.756274 | 0.80 |
| DUF3697 | 2 | UBAP2, UBAP2L | 0.728186 | 0.79 |
| DUF4537 | 2 | C11orf16, VWA3B | 0.671401 | 0.79 |
| DUF1604 | 1 | GPATCH1 | 0.663934 | 0.79 |
| DUF4210 | 2 | FAM214A, FAM214B | 0.661396 | 0.79 |
| DUF1232 | 1 | RNF170 | 0.649466 | 0.77 |
| DUF1674 | 1 | SDHAF4 | 0.640992 | 0.77 |

| | | | | |
|---|---|---|---|---|
| **peptide** | | | | |
| DUF902 | 2 | CREBBP, EP300 | 0.735166 | 0.71 |
| DUF1242 | 2 | TMEM167A, TMEM167B | 0.729637 | 0.71 |
| DUF4339 | 1 | TDNAJC13 | 0.655891 | 0.71 |
| DUF3652 | 1 | HTT | 0.626383 | 0.70 |
| DUF3342 | 5 | KIAA1841, ZBTB34, ZBTB37, ZBTB46, ZBTB8A | 0.603940 | 0.69 |
| DUF1674 | 1 | SDHAF4 | 0.596013 | 0.69 |
| DUF2205 | 1 | SCOC | 0.591663 | 0.69 |
| DUF3337 | 1 | WDR48 | 0.591186 | 0.69 |
| DUF3697 | 2 | UBAP2, UBAP2L | 0.580904 | 0.68 |
| DUF3657 | 2 | FAM135A, FAM135B | 0.577026 | 0.68 |
| DUF4596 | 2 | PRR36 | 0.565096 | 0.68 |
| DUF3677 | 1 | INTS1 | 0.53303 | 0.52 |
| DUF1232 | 1 | RNF170 | 0.532215 | 0.51 |
| DUF4723 | 1 | C9orf57 | 0.505842 | 0.42 |
| DUF4801 | 1 | MGA | 0.501839 | 0.39 |
| **small molecule** | | | | |
| DUF4518 | 1 | C3orf38 | 0.798212 | 0.57 |
| DUF4461 | 1 | TCAIM | 0.778624 | 0.57 |
| DUF4559 | 1 | CXorf38 | 0.772098 | 0.57 |
| DUF4569 | 1 | CXorf21 | 0.770323 | 0.57 |
| DUF4689 | 1 | C16orf54 | 0.769033 | 0.57 |
| DUF938 | 1 | METTL26 | 0.768819 | 0.57 |
| DUF4657 | 2 | AC037459.1, C8orf58 | 0.768276 | 0.57 |
| DUF4628 | 1 | C1orf226 | 0.765329 | 0.57 |
| DUF2462 | 1 | C19orf53 | 0.761421 | 0.57 |
| DUF2048 | 1 | ABHD18 | 0.750025 | 0.57 |
| DUF4471 | 1 | DNAAF3 | 0.748775 | 0.57 |
| DUF2475 | 3 | C2orf70, FAM166A, FAM166B | 0.746606 | 0.57 |
| DUF4564 | 1 | C17orf62 | 0.744047 | 0.57 |
| DUF4609 | 1 | SPATA33 | 0.74339 | 0.57 |
| DUF814 | 2 | CCDC25, NEMF | 0.743034 | 0.57 |
| DUF1053 | 6 | ADCY2, ADCY4, ADCY5, ADCY6 ADCY7, ADCY8 | 0.742931 | 0.57 |
| DUF4464 | 1 | C4orf22 | 0.740633 | 0.57 |
| DUF1736 | 4 | TMTC1, TMTC2, TMTC3, TMTC4 | 0.740331 | 0.57 |
| DUF4662 | 1 | C22orf31 | 0.738987 | 0.57 |
| DUF4572 | 1 | C9orf135 | 0.738065 | 0.57 |

| | | | | |
|---|---|---|---|---|
| DUF4542 | 1 | ENSG00000214556 | 0.736131 | 0.57 |
| DUF2228 | 1 | HPF1 | 0.732842 | 0.57 |
| DUF4699 | 1 | C4orf19 | 0.731914 | 0.57 |
| DUF4481 | 1 | TMEM268 | 0.72959 | 0.57 |
| DUF167 | 1 | C15orf40 | 0.72937 | 0.57 |
| DUF2431 | 3 | FDXACB1 | 0.728382 | 0.57 |
| DUF1986 | 33 | CTRL, F7, GZMK, KLK13, KLK6, LPA, OVCH1, PLG, PRSS22, PRSS27, PRSS36, PRSS38, PRSS42, PRSS45, PRSS46P, PRSS48, PRSS50, PRSS55, PRSS56, PRSS8, PRTN3, TMPRSS11D, TMPRSS11E, TMPRSS11F, TMPRSS13, TMPRSS15, TMPRSS2, TMPRSS3, TMPRSS6, TMPRSS9 | 0.727394 | 0.57 |
| DUF4539 | 1 | C17orf53 | 0.727391 | 0.57 |
| DUF4637 | 2 | ENSG00000154768 | 0.726993 | 0.57 |
| DUF829 | 1 | TMEM53 | 0.724484 | 0.57 |
| DUF4553 | 1 | ENSG00000155640 | 0.722978 | 0.57 |
| DUF4147 | 1 | GLYCTK | 0.721339 | 0.57 |
| DUF4504 | 1 | C1orf74 | 0.720823 | 0.57 |

**Table S5: Whole-domain prediction of which ligands DUFs bind.** DUFs with a prediction score of at least 0.48, 0.49, 0.63, 0.49, and 0.72 for RNA, DNA, ion, peptide and small molecule, respectively; these predictions score thresholds correspond to a global recall of 0.5, as estimated in cross-validation testing. For each DUF, we list the number of domain instances across the human genome, the names of all the human genes it was identified in, the dSPRINT prediction score for binding the ligand, and an estimated precision. We calculate the estimated precision for each ligand-binding prediction score by analyzing performance in cross-validation testing as follows. Since precision on our training set is not strictly monotonic with respect to the predicted binding score (i.e., it does not always go down as the prediction score goes down), we rank prediction scores from largest and smallest and for each prediction score take the median measured precision score across a window of size seven. Then, for a particular predicted binding score, we take the minimum precision of all the binding scores larger than or equal to it, to conservatively enforce monotonicity.
